# Glucopyranosylidene-Spiro-Thiazolinones: Synthetic Studies and Determination of Absolute Configuration by TDDFT-ECD Calculations

**DOI:** 10.3390/molecules22101760

**Published:** 2017-10-19

**Authors:** Katalin E. Szabó, Sándor Kun, Attila Mándi, Tibor Kurtán, László Somsák

**Affiliations:** Department of Organic Chemistry, University of Debrecen, P.O. Box 400, H-4002 Debrecen, Hungary; szabo.erzsebet.katalin@science.unideb.hu (K.E.S.); kun.sandor@science.unideb.hu (S.K.); mandi.attila@science.unideb.hu (A.M.); kurtan.tibor@science.unideb.hu (T.K.)

**Keywords:** spiro compound, thiazolinone, glucose derivative, TDDFT-ECD

## Abstract

Reactions of *O*-peracylated *C*-(1-bromo-β-d-glucopyranosyl)formamides with thioamides furnished the corresponding glucopyranosylidene-spiro-thiazolin-4-one. While *O*-debenzoylations under a variety of conditions resulted in decomposition, during *O*-deacetylations the addition of MeOH to the thiazolinone moiety was observed, and with EtOH and water similar adducts were isolated or detected. The structure and stereochemistry of the new compounds were established by means of NMR and electronic circular dichroism (ECD) data supported by time-dependent density functional theory ECD (TDDFT-ECD) calculations. TDDFT-ECD calculations could efficiently distinguish the proposed epimeric products having different absolute configuration in the spiro heterocyclic ring.

## 1. Introduction

During the past two decades, several glucopyranosylidene-spiro heterocycles have emerged as potent inhibitors of glycogen phosphorylase (GP) [1,2,3,4,5]. The liver isoform of GP is a validated target in the search for new therapeutic possibilities against type 2 diabetes mellitus [6], nevertheless, its inhibition is considered to have potential in other important pharmacological fields such as possible medication and/or prevention of cardiovascular disorders [7,8], cerebral ischemias [9,10] and tumorous growth [11,12,13,14,15,16].

**Chart 1:**
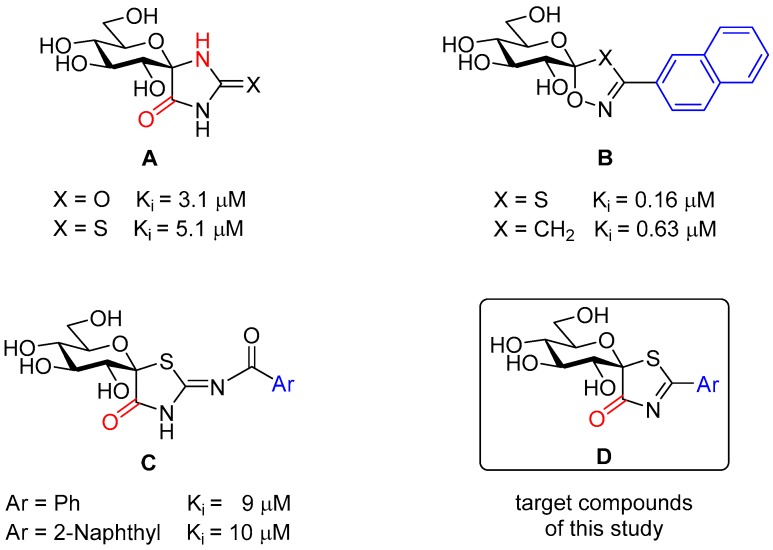
Selected spirobicyclic inhibitors of rabbit muscle glycogen phosphorylase **A**, **B** and **C** (K_i_ dissociation constant of the enzyme-inhibitor complex) and the target compounds of the present study (**D**).

The first spirocyclic GP inhibitor (GPI), a glucopyranosylidene-spiro-hydantoin (**A**, X = O in Chart 1) was disclosed in the mid-nineties [17] followed by the more easily synthesized thiohydantoin counterpart (**A**, X = S) [18,19]. Both compounds were active as GPIs in the low micromolar concentration range. Glucopyranosylidene-spiro-oxathiazoles (**B**, X = S) [20,21] and isoxazolines (**B**, X = CH_2_) [22] identified some ten years later proved to be submicromolar GPIs and still belong in the top ten most efficient glucose-derived inhibitors of the enzyme. For the strong binding of compounds **A** to the enzyme, X-ray crystallography of the enzyme-inhibitor complexes revealed the importance of the H-bond donor β-NH, as well as that of the α-carbonyl groups highlighted in red [23,24]. Similar studies with compounds **B** concluded that bulky substituents such as the 2-naphthyl moiety (marked in blue) better contributed to the binding even in the absence of the hydrogen bonding β-NH and the carbonyl in the α-position [22]. An attempt was made to unify these properties by the study of compounds **C** [3]. Surprisingly, these derivatives proved rather weak inhibitors and this was attributed to the =N–C=O linker between the spirocycle and the aryl groups resulting in an unfavorable orientation of the latter for the binding. To circumvent this problem the synthesis of compounds **D** was envisaged, and the results of these studies are presented in this paper.

## 2. Results and Discussion

In our previous study dealing with the preparation of glucopyranosylidene-spiro-iminothiazolidinone **C** [3] bromo-amide derivative **1** was found to be the substrate of choice in reactions with thiourea, therefore its transformation with thiobenzamide was investigated first (Table 1) to find the best conditions for providing the present target compounds. In refluxing EtOH, that was advantageous earlier, only **6** [25] could be obtained (entry 1). Since acetone also proved to be a suitable solvent previously, the higher boiling diethylketone was tried next, but **7** [26] could be isolated as the only product (entry 2). Much less time was needed for the complete consumption of the starting **1** in pyridine or DMF (entries 3 and 4), however, only the formation of **8** [27] or **9** [28] was observed, respectively. Following this, less reactive solvents such as toluene and *m*-xylene (entries 5 and 6) were tested affording similar yields of the expected compound **2** with a significantly shorter reaction time for the latter. In ether type solvents (entries 7–9) and nitromethane (entry 10) the yields of **2** remained the same, therefore, the use of *m*-xylene was further optimized. Applying an inert atmosphere (entry 11) or addition of a silver salt (entry 12) left the yield practically unchanged, while microwave heating resulted in a considerable increase in the yield of **2** (entry 13). This was further improved by raising the temperature (entry 14), however even these conditions did not allow diminishing the amount of thiobenzamide (entry 15). Other thioamides gave the expected spiro derivatives **3**–**5** in acceptable yields (entries 16–18). The formation of the depicted molecules **2**–**5** with an inversion of configuration at the newly formed spiro carbon is consistent with a presumable bimolecular nucleophilic substitution mechanism and concurs with earlier observations on the stereochemical outcome of the synthesis of glucopyranosylidene-spiro-iminothiazolidinone **C** [3].

Attempts to remove the benzoyl protecting groups [29] under a series of base (NaOMe/MeOH, 1 day; KCN MeOH, 4 weeks decomposition) or acid catalyzed (AcCl/MeOH, 4 weeks no reaction) transesterification conditions or under basic hydrolysis conditions (4 equiv. LiOH/MeOH, 0 °C decomposition) proved unsuccesful.

**Scheme 1:**
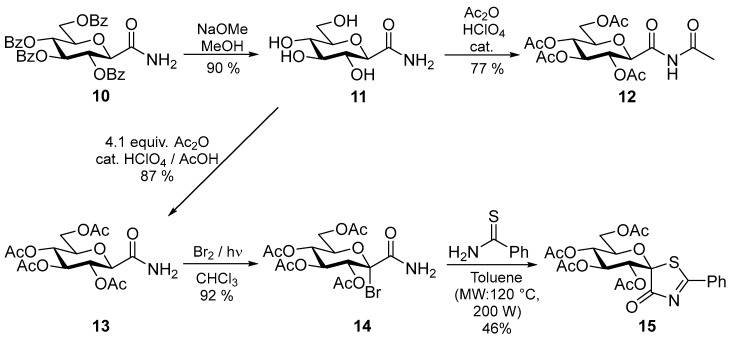
Synthesis of spiro-thiazolinone **15** with *O*-acetyl protecting groups.

After this experience the use of the more readily removable *O*-acetyl protecting groups was envisaged. To this end, the *O*-benzoyl groups of **10** [28] were removed by using the Zemplén protocol and the resulting amide **11** was treated with Ac_2_O under acidic catalysis (Scheme 1). Since *N*-acetylation also took place with an excess of the acetylating agent to give **12**, four equivalents of Ac_2_O in AcOH had to be used to yield the expected **13** [30]. This compound was subjected to radical-mediated bromination conditions [31,32] to afford **14** [19] which could be transformed to thiazolinone **15** with thiobenzamide. It is to be noted, that in this latter transformation the conditions used to get the *O*-perbenzoylated **2**–**5** resulted in only 14–18% yields due to decomposition, but at lower temperature in toluene 46% of **15** could be achieved.

For the removal of the *O*-acetyl protective groups [29] of **15** a wide range of conditions recommended in the literature were attempted (Table 2). Under classical Zemplén conditions or transesterifications catalyzed by K_2_CO_3_ or KCN (entries 1–3, respectively) only the formation of multicomponent mixtures could be observed. Similar outcomes were achieved by using nucleophilic ester cleaving reagents (entries 4 and 5) or the non-nucleophilic base DBU (entry 6). However, LiOH in MeOH (entry 7) gave a mixture of **16** and **17**, and these compounds were obtained in better yields with a substoichiometric amount of LiOH (entry 8). From the attempted acid catalyzed transesterification conditions (entries 9–11), it was only the use of KHSO_4_ (entry 11) that resulted in a moderate yield of **18** with the 2’-*O*-acetyl group remaining in the molecule.

The presence of the MeOH addition products **17** and **18** in the deacetylation mixtures prompted us to investigate the reaction of **2** and **15** with MeOH and EtOH without any other additive (Table 3). It turned out that both alcohols added to the thiazolinone moiety to furnish **19**–**22** (entries 1–4), respectively, as the major stereoisomers indicated by the characteristic signals in the NMR spectra (see detailed structural elucidation below). Removal of the solvent alcohol at 30 °C allowed us to characterize these addition products. The presence of minor components (<10%) could be observed in the ^1^H-NMR spectra (see Supporting Information), however, their separation and purification proved infeasible due to the same chromatographic mobility of the substances. Therefore, the formation of both possible diastereomeric addition products cannot be excluded. This addition reaction proved to be reversible since by heating of **19** (at 80 °C) or **21** (at 60 °C) in toluene thiazolinones **2** and **15** were recovered, respectively. To the best of our knowledge, similar alcohol addition was reported only for 2-perfluoroalkyl-5-methoxycarbonylmethylene-thiazoline-4-one type compounds [33,34]. Our findings indicate that the presence of strongly electron withdrawing substituents, such as the perfluoroalkyl groups are not required for the ROH addition.

The addition of water was also tested with **15** (entry 5) and the formation of **23** was observed in a mixture with the starting compound (**15**:**23**~1:0.4). The appearance of **23** made it clear that a similar addition may occur during the enzymatic tests necessarily carried out under aqueous conditions, thereby thwarting the study of these compounds as enzyme inhibitors.

**Chart 2:**
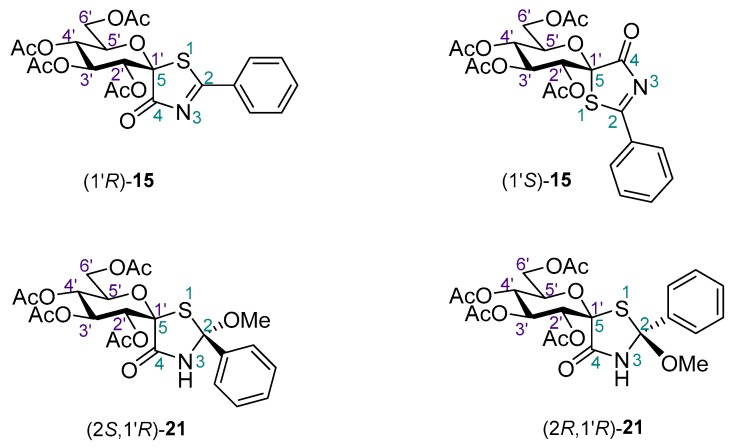
Possible stereoisomers of **15** and **21**.

Structural elucidation of the *O*-perbenzoylated spirocycles **2**–**5** was carried out by NMR and MS methods. The ^13^C-NMR spectra contained signals in the range of 194–195 ppm and 187–188 ppm for C-2 and C-4, respectively, while similar resonances for **15** appeared at 194.7 ppm (C-2) and 187.0 ppm (C-4) (for numbering see Chart 2). Molecular masses determined by MS corroborated the formation of the spirocycles **2**–**5** and **15**. The configuration of the 1’,5-spiro carbon in **2** was established by determining the three-bond heteronuclear coupling constant between the axial H-2’ (^4^*C*_1_ conformation of the sugar ring followed from the vicinal proton–proton couplings in the ^1^H-NMR spectra, see experimental) and C-4. The observed 6 Hz value indicated *trans* diaxial arrangement of these nuclei thereby proving the (*R*) configuration of the spiro center. For **3**–**5**, the similarities of the chemical shifts for the sugar ring protons lead to the assumption that the spiro configuration is the same.

Both ^1^H- and ^13^C-NMR spectra of **17**–**22** exhibited characteristic signals for the OMe and OEt groups (see experimental section for specifics). The ^1^H-NMR spectra showed resonances for the exchangable NH protons (6.6–7.2 ppm), and in the ^13^C-NMR spectra signals appeared for C-2 in the range of 90–100 ppm instead of the 194–195 ppm resonances of the starting materials.

Since no NMR method seemed suitable to determine the configuration of C-2 in the addition products **17**–**22**, ECD measurements and TDDFT-ECD calculations were also performed for **21**. These studies were extended to **15** in order to confirm the NMR results independently (Chart 2 shows the studied stereoisomeric structures).

**Figure 1:**
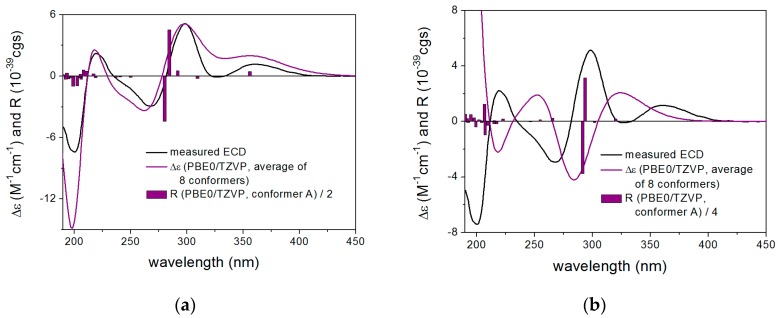
Experimental ECD spectrum of **15** in MeCN compared with the Boltzmann-weighted PBE0/TZVP PCM/MeCN ECD spectra of (**a**) (1’*R*)-**15** and (**b**) (1’*S*)-**15** both computed for the ωB97XD/TZVP PCM/MeCN conformers. The bars represent the rotational strength values of the lowest-energy conformers.

For the determination of the absolute configuration of the C-1’ chirality center, the solution TDDFT-ECD method was utilized on the (1’*R*) and (1’*S*) diastereomers of **15** (Chart 2) [35,36]. Based on the literature data for other acetylated glucopyranose derivatives, the ECD spectrum was expected to be governed by the aglycon part containing the C-1’ chirality center as part of a 2-phenylthiazolin-4-one unit [37]. The initial conformers generated for both stereoisomers with the OPLS-2005 force field were reoptimized at the B3LYP/6-31G(d) in vacuo and the ωB97XD/TZVP [35,38] PCM/MeCN levels (see Supporting Information). ECD calculations were performed with various functionals (B3LYP, BH&HLYP, CAM-B3LYP and PBE0) and the TZVP basis set for the low-energy conformers over 1% Boltzmann population. The computed averaged ECD spectrum of the (1’*R*) diastereomer reproduced all the transitions of the experimental ECD spectrum well (Figure 1a). In contrast, the (1’*S*) diastereomer had a much poorer mirror-image agreement of the experimental Cotton effects (CEs) below 325 nm (Figure 1b) confirming the decisive role of the C-1’ chirality center in the ECD pattern. The highest-wavelength positive n-π* CE could not be reproduced by the computed ECD of the (1’*S*) epimer, since similar to the other transitions, opposite CE should have been calculated. Based on the good agreement of the (1’*R*) diastereomer, the (1’*R*) absolute configuration of **15** was unambiguously determined in concord with the NMR analysis of **2**.

**Figure 2:**
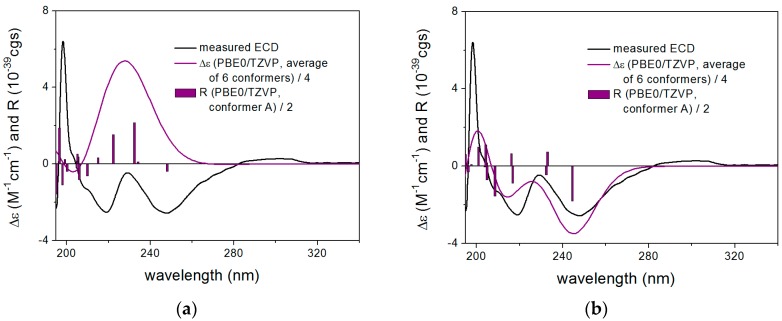
Experimental ECD spectrum of **21** in MeCN compared with the Boltzmann-weighted PBE0/TZVP PCM/MeCN ECD spectra of (**a**) (2*R*,1’*R*)-**21** and (**b**) (2*S*,1’*R*)-**21** both computed for the ωB97XD/TZVP PCM/MeCN conformers. The bars represent the rotational strength values of the lowest-energy conformers.

In the case of **21**, both the C-1’ and the C-2 chirality centers were expected to contribute to the ECD spectrum and ECD calculations were performed for the (2*R*,1’*R*) and (2*S*,1’*R*) diastereomers (Chart 2). The computed ECD spectra of (2*R*,1’*R*)-**21** and (2*S*,1’*R*)-**21** showed opposite CEs in the 190–290 nm spectral range with acceptable mirror image agreement (Figure 2a) or good reproduction of the experimental ECD spectrum (Figure 2b), respectively. However, the computed ECD of (2*S*,1’*R*)-**21** had two well-separated negative CEs, the shape of which were much more similar to the experimental ECD than that of the broad positive computed CE of (2*R*,1’*R*)-**21**. Thus, the good overall agreement of (2*S*,1’*R*)-**21** allowed the unambiguous elucidation of the absolute configuration at C-2 as (*S*).

Compounds **19**–**22** were formed as preponderant diastereomers (e.g., (2*S*,1’*R*)-**21** in Chart 2) although nucleophilic attack of the alcohol could have been expected from both sides of the planar thiazolinone ring. This observation may be explained by a steric hindrance of the C-2 center by the 2’-OAc or 2’-OBz groups facilitating the addition reaction from the opposite side. This can be visualized by the computed conformers of (1’*R*)-**15** (Figure S1b in the Supporting Information).

## 3. Conclusions

Glucopyranosylidene-spiro-thiazolinones were prepared in *O*-perbenzoylated and *O*-peracetylated forms by reacting the corresponding *C*-(1-bromo-β-d-glucopyranosyl)formamides with arenethiocarboxamides in *m*-xylene under microwave heating conditions. While *O*-debenzoylations could not be achieved, during the *O*-deacetylation by the Zemplén protocol addition of MeOH onto the thiazolinone moiety was observed. This addition could be extended to EtOH and even water and the reactions proved to be reversible. Electronic circular dichroism spectroscopy aided by TDDFT-ECD computations could differentiate the possible diastereomers and confirmed the absolute configuration assigned by NMR analysis of the new compounds and also allowed the determination of the absolute configuration of the new stereogenic center formed upon the addition of alcohols. The formation of the water addition product under aqueous conditions prevented the test of these compounds as enzyme inhibitors.

## 4. Experimental

### 4.1. General Methods

Melting points were measured on a Kofler hot-stage and are uncorrected. Optical rotations were determined with a Perkin-Elmer 241 polarimeter at r.t. NMR spectra and were recorded with Bruker 360 (360/90 MHz for ^1^H/^13^C) or Bruker 400 (400/100 MHz for ^1^H/^13^C) or Avance DRX 500 (500/125 MHz for ^1^H/^13^C) spectrometers (Bruker, Karlsruhe, Germany; Billerica, MA, USA). Chemical shifts are referenced to the internal TMS (^1^H), or to the residual solvent signals (^13^C). In the NMR spectra complete signal assignments are based on COSY, HSQC, and HSQMBC correlations. Mass spectra were obtained by a Bruker micrOTOF-Q instrument. TLC was performed on DC-Alurolle Kieselgel 60 F_254_ (Merck, Darmstadt, Germany), and the plates were visualised under UV light and by gentle heating (generally no spray reagent was used but, if more intense charring was necessary, the plate was sprayed with the following solution: abs. EtOH (95 mL), cc.H_2_SO_4_ (5 mL) anisaldehyde (1 mL)). For column chromatography Kieselgel 60 (Merck, particle size 0.063–0.200 mm) was used. Organic solutions were concentrated in vacuo at 40–60 °C (water bath). Xylene and toluene were distilled from P_4_O_10_ and stored over sodium wires. MeOH was purified by distillation after refluxing for a couple of hours with magnesium turnings and iodine. CHCl_3_ was distilled from P_4_O_10_ and stored over 4Å molecular sieves. Thioamides were purchased from Sigma-Aldrich (Steinheim, Germany). *C*-(2,3,4,6-tetra-*O*-benzoyl-1-bromo-β-d-glucopyranosyl)formamide (**1**) and *C*-(2,3,4,6-tetra-*O*-benzoyl-β-d-glucopyranosyl)formamide (**10**) were synthesized according to published procedures [28]. Microwave-assisted reactions were carried out using a CEM-Discover Focused microwave synthesis system (2450 MHz).

General procedure I for the synthesis of *(1’R)-1’,5’-anhydro-2’,3’,4’,6’-tetra-O-benzoyl-d-glucitol-spiro-*[1’,5]*-2-arylthiazolin-4-ones* (**2**–**5**). *C*-(2,3,4,6-Tetra-*O*-benzoyl-1-bromo-1-deoxy-β-d-glucopyranosyl)formamide (**1**) [28] and the corresponding thioamide (2.0 equiv.) were mixed in dry xylene (10 mL/mmol), in a sealed vial and the reaction mixture was stirred for 1.5 h at 140 °C (200 W). Then the mixture was evaporated, and the residue was purified by column chromatography.

General procedure II for the preparation of *(2S,1’R)-2’,3’,4’,6’-tetra-O-acyl-1’,5’-anhydro-d-glucitol-spiro-*[1’,5]*-2-alkoxy-2-phenylthiazolidin-4-ones* (**19**–**22**)*.* The peracetylated or the perbenzoylated spiro thiazolinone (**2** or **15**) was dissolved in the corresponding alcohol (30 mL/mmol) and the solution was stirred at rt for ~1 day. When the starting material was consumed (TLC, 1:3 EtOAc/toluene) the solvent was evaporated at diminished pressure and 30 °C water bath temp.

### 4.2. Characterization of the Compounds

*(1’R)-1’,5’-Anhydro-2’,3’,4’,6’-tetra-O-benzoyl-d-glucitol-spiro-[1’,5]-2-phenylthiazolin-4-one* (**2**). Prepared from compound **1** (0.50 g, 0.71 mmol) and thiobenzamide (0.19 g, 1.42 mmol) according to general procedure I. Purified by column chromatography (1:5 EtOAc/hexane) to give 0.28 g (53%) orange amorphous solid. *R*_f_ = 0.42 (2:3 EtOAc/hexane); [α]_D_ +87 (c 0.50, CHCl_3_). ^1^H-NMR (400 MHz, CDCl_3_) δ (ppm): 8.09–7.93 (6H, m, aromatics), 7.86–7.78 (4H, m, aromatics), 7.60 (1H, t, *J* = 7.5 Hz, aromatics), 7.55–7.31 (10H, m, aromatics), 7.26–7.20 (4H, m, aromatics), 6.86 (1H, pseudo t, *J* = 9.7 Hz, H-3’), 6.33 (1H, d, *J* = 9.7 Hz, H-2’), 5.95 (1H, pseudo t, *J* = 10.0 Hz, H-4’), 5.39 (1H, ddd, *J* = 10.1, 4.1, 2.3 Hz, H-5’), 4.67 (1H, dd, *J* = 12.6, 2.3 Hz, H-6’a), 4.51 (1H, dd, *J* = 12.6, 4.1 Hz, H-6’b); ^13^C-NMR (100 MHz, CDCl_3_) δ (ppm): 195.3 (C=N), 187.7 (CON, ^3^*J*_H-2’,CON_ = 6.0 Hz, from HSQMBC at 125 MHz), 166.1, 166.3, 165.2, 164.8 (C=O), 136.3–127.9 (aromatics), 94.5 (C-1’), 71.8 (C-5’), 70.74, 70.65 (C-2’, C-3’), 68.6 (C-4’), 62.6 (C-6’). ESI-MS positive mode (*m*/*z*): calcd for C_42_H_31_NNaO_10_S ([M + Na]^+^): 764.156. Found: 764.155.

*(1’R)-1’,5’-Anhydro-2’,3’,4’,6’-tetra-O-benzoyl-d-glucitol-spiro-[1’,5]-2-(1-naphthyl)thiazolin-4-one* (**3**). Prepared from compound **1** (0.40 g, 0.57 mmol) and naphthalene-1-carbothioamide (0.21 g, 1.14 mmol) according to general procedure I. Purified by column chromatography (1:5 EtOAc/hexane) to give 0.15 g (32%) orange amorphous solid. *R*_f_ = 0.40 (2:3 EtOAc/hexane); [α]_D_ +107 (c 0.47, CHCl_3_); ^1^H-NMR (400 MHz, CDCl_3_) δ (ppm): 9.06 (1H, d, *J* = 8.6 Hz, aromatics), 8.09–7.97 (6H, m, aromatics), 7.88–7.81 (4H, m, aromatics), 7.62–7.33 (12H, m, aromatics), 7.28–7.20 (4H, m, aromatics), 6.86 (1H, pseudo t, *J* = 9.7 Hz, H-3’), 6.35 (1H, d, *J* = 9.7 Hz, H-2’), 5.96 (1H, pseudo t, *J* = 10.0 Hz, H-4’), 5.45 (1H, ddd, *J* = 10.1, 4.2, 2.6 Hz, H-5’), 4.69 (1H, dd, *J* = 12.6, 2.6 Hz, H-6’a), 4.52 (1H, dd, *J* = 12.6, 4.2 Hz, H-6’b); ^13^C-NMR (100 MHz, CDCl_3_) δ (ppm): 195.6 (C=N), 188.1 (CON), 166.2, 165.4, 165.3, 164.9 (C=O), 136.6–124.6 (aromatics), 93.8 (C-1’), 71.9 (C-5’), 70.9, 70.7 (C-2’, C-3’), 68.7 (C-4’), 62.7 (C-6’). ESI-MS positive mode (*m*/*z*): calcd for C_46_H_33_NNaO_10_S ([M + Na]^+^): 814.172. Found: 814.171.

*(1’R)-1’,5’-Anhydro-2’,3’,4’,6’-tetra-O-benzoyl-d-glucitol-spiro-[1’,5]-2-(2-naphthyl)thiazolin-4-one* (**4**). Prepared from compound **1** (0.50 g, 0.71 mmol) and naphthalene-2-carbothioamide (0.27 g, 1.42 mmol) according to general procedure I. Purified by column chromatography (1:4 EtOAc/hexane) to give 0.23 g (40%) orange amorphous solid. *R*_f_ = 0.40 (2:3 EtOAc/hexane); [α]_D_ +62 (c 0.63, CHCl_3_); ^1^H-NMR (400 MHz, CDCl_3_) δ (ppm): 8.55 (1H, s, aromatics), 8.08–7.97 (5H, m, aromatics), 7.89–7.78 (6H, m, aromatics), 7.62–7.30 (11H, m, aromatics), 7.27–7.19 (4H, m, aromatics), 6.89 (1H, pseudo t, *J* = 9.7 Hz, H-3’), 6.36 (1H, d, *J* = 9.7 Hz, H-2’), 5.97 (1H, pseudo t, *J* = 10.0 Hz, H-4’), 5.42 (1H, ddd, *J* = 10.1, 4.1, 2.5 Hz, H-5’), 4.69 (1H, dd, *J* = 12.6, 2.5 Hz, H-6’a), 4.51 (1H, dd, *J* = 12.6, 4.1 Hz, H-6’b); ^13^C-NMR (100 MHz, CDCl_3_) δ (ppm): 194.9 (C=N), 187.6 (CON), 166.1, 165.4, 165.3, 165.0 (C=O), 137.1–123.9 (aromatics), 94.6 (C-1’), 71.9 (C-5’), 70.8, 70.7 (C-2’, C-3’), 68.7 (C-4’), 62.7 (C-6’). ESI-MS positive mode (*m*/*z*): calcd for C_46_H_33_NNaO_10_S ([M + Na]^+^): 814.172. Found: 814.169.

*(1’R)-1’,5’-Anhydro-2’,3’,4’,6’-tetra-O-benzoyl-d-glucitol-spiro-[1’,5]-2-(4-methylphenyl)thiazolin-4-one* (**5**). Prepared from compound **2** (0.40 g, 0.57 mmol) and 4-methylbenzenethioamide (0.17 g, 1.14 mmol) according to general procedure I. Purified by column chromatography (1:4 EtOAc/hexane) to give 0.23 g (53%) orange amorphous solid. *R*_f_ = 0.31 (1:2 EtOAc/hexane); [α]_D_ +87 (c 0.61, CHCl_3_); ^1^H-NMR (360 MHz, CDCl_3_) δ (ppm): 8.08–8.03 (2H, m, aromatics), 8.00–7.89 (4H, m, aromatics), 7.85–7.77 (4H, m, aromatics), 7.60–7.30 (9H, m, aromatics), 7.27–7.20 (5H, m, aromatics), 6.83 (1H, pseudo t, *J* = 9.7 Hz, H-3’), 6.29 (1H, d, *J* = 9.7 Hz, H-2’), 5.92 (1H, pseudo t, *J* = 9.9 Hz, H-4’), 5.37 (1H, ddd, *J* = 10.0, 4.0, 2.3 Hz, H-5’), 4.65 (1H, dd, *J* = 12.6, 2.3 Hz, H-6’a), 4.48 (1H, dd, *J* = 12.6, 4.0 Hz, H-6’b), 2.38 (3H, s, CH_3_); ^13^C-NMR (90 MHz, CDCl_3_) δ (ppm): 194.9 (C=N), 187.8 (CON), 166.1, 166.4, 165.3, 164.9 (C=O), 148.3–128.0 (aromatics), 94.4 (C-1’), 71.8 (C-5’), 70.8, 70.7 (C-2’, C-3’), 68.7 (C-4’), 62.7 (C-6’), 22.1 (CH_3_). ESI-MS positive mode (*m*/*z*): calcd for C_43_H_33_NNaO_10_S ([M + Na]^+^): 778.172. Found: 778.171.

*C-(β-D-Glucopyranosyl)formamide* (**11**). *C*-(2,3,4,6-tetra-*O*-benzoyl-β-d-glucopyranosyl) formamide [28] (**10**, 5.0 g, 8.03 mmol) was dissolved in dry MeOH (100 mL) and 10 drops of a 1 M methanolic NaOMe solution were added. The reaction mixture was stirred at r.t. and monitored by TLC (3:7 MeOH/CHCl_3_, ~1 day). After complete conversion of **10** the mixture was neutralised with acetic acid and the product precipitated as a white solid. The material was filtered off (1.20 g). The filtrate was concentrated and the residue was crystallized again from methanol to give 0.30 g of **11**. Yield: 90%. M.p.: 204–206 °C; *R*_f_ = 0.15 (1:2 MeOH/CHCl_3_); [α]_D_ +30 (c 0.53, H_2_O); ^1^H-NMR (400 MHz, D_2_O) δ (ppm): 3.95–3.85 (2H, m, H-1 and/or H-2 and/or H-3 and/or H-4 and/or H-5 and/or H-6a), 3.76 (1H, dd, *J* = 12.2, 4.0 Hz, H-6b), 3.60–3.41 (4H, m, H-1 and/or H-2 and/or H-3 and/or H-4 and/or H-5 and/or H-6a); ^13^C-NMR (100 MHz, D_2_O) δ (ppm): 174.7 (C=O), 80.0, 78.6, 77.5, 72.3, 69.8 (C-1–C-5), 61.4 (C-6). ESI-MS positive mode (*m*/*z*): calcd for C_7_H_13_NNaO_6_S ([M + Na]^+^): 230.064. Found: 230.063.

*N-Acetyl-C-(2,3,4,6-tetra-O-acetyl-β-d-glucopyranosyl)formamide* (**12**). Compound **11** (0.30 g, 1.45 mmol) was dissolved in acetic anhydride (5 mL, 52.89 mmol) and one drop of HClO_4_ was added and the mixture was stirred at rt for 1 h. The resulting solution was poured into ice-water (50 mL), which was then extracted with CHCl_3_ (3 × 20 mL). The unified CHCl_3_ phases were washed with NaHCO_3_ (2 × 20 mL), then with water (1 × 20 mL). The organic phase was dried over MgSO_4_, concentrated under diminished pressure, and the residual syrup crystallized on addition of diethyl ether to give 0.46 g (77%) white solid. M.p.: 135–137 °C; *R*_f_ = 0.34 (1:1 Acetone/hexane); [α]_D_ +8 (c 0.59, CHCl_3_); ^1^H-NMR (360 MHz, CDCl_3_) δ (ppm): 8.78 (1H, s, NH), 5.30 (1H, pseudo t, *J* = 9.3 Hz, H-2 or H-3 or H-4), 5.19–5.07 (2H, m, H-2 and/or H-3 and/or H-4), 4.29 (1H, dd, *J* = 12.5, 4.7 Hz, H-6a ), 4.17 (1H, dd, *J* = 12.5, 1.1 Hz, H-6b), 4.00 (1H, d, *J* = 9.8 Hz, H-1), 3.82 (1H, ddd, *J* = 9.8, 4.7, 1.1 Hz, H-5), 2.42 (3H, s, NHCOC*H*_3_), 2.12, 2.07, 2.05, 2.03 (4 × 3H, 4 s, CH_3_); ^13^C-NMR (90 MHz, CDCl_3_) δ (ppm): 171.9, 170.6, 170.0, 169.7, 169.4, 166.0 (C=O), 76.4, 76.0, 73.0, 68.9, 67.8 (C-1–C-5), 61.8 (C-6), 25.4 (NHCO*C*H_3_), 20.8, 20.6 × 3 (4 × CH_3_). ESI-MS positive mode (*m*/*z*): calcd for C_17_H_23_NNaO_11_ ([M + Na]^+^): 440.116. Found: 440.116.

*C-(2,3,4,6-Tetra-O-acetyl-β-d-glucopyranosyl)formamide* (**13**). Compound **11** (1.50 g, 7.24 mmol) was suspended in glacial acetic acid (20 mL) and acetic anhydride (2.8 mL, 29.68 mmol) and one drop of HClO_4_ were added. The reaction mixture was stirred at room temperature. After disappearance of the starting material (TLC, 2:1 Acetone/hexane, 2 h) the resulting solution was poured into ice-water (100 mL), which was then extracted with CHCl_3_ (3 × 50 mL). The unified CHCl_3_ phases were washed with NaHCO_3_ (2 × 50 mL), then with water (1 × 50 mL). The organic phase was dried over MgSO_4_, concentrated under diminished pressure, and the residue was crystallized from ether–hexane mixture to give 2.35 g (87%) of **13**. The compound characterization data are identical with those reported [30].

*C-(2,3,4,6-Tetra-O-acetyl-1-bromo-β-d-glucopyranosyl)formamide* (**14**). Compound **13** (2.30 g, 6.10 mmol) was dissolved in anhydrous CHCl_3_ (130 mL) and bromine (1 mL, 19.5 mmol) and some K_2_CO_3_ were added (as the acid scavenger). The mixture was placed in an Erlenmeyer flask above a heat lamp (375 W, white, distance from the lamp ~1–2 cm) and refluxed until TLC showed complete transformation (1:1 Acetone/hexane, ~2 h). It was then washed with 5% aq. Na_2_SO_3_ (3 × 30 mL) and NaHCO_3_ (2 × 30 mL) solutions. Drying (MgSO_4_) and evaporation of the solvent gave 2.56 g (92%) yellowish foam, which was sufficiently pure for the nextstep. The compound characterization data are identical with those reported [19].

*(1’R)-1’,5’-Anhydro-2’,3’,4’,6’-Tetra-O-acetyl-d-glucitol-spiro-[1’,5]-2-phenylthiazolin-4-one* (**15**). Compound **14** (0.35 g, 0.77 mmol) and thiobenzamide (0.21 g, 1.54 mmol) were mixed in dry toluene (3 mL) in a sealed vial and the reaction mixture was stirred for 2 h at 120 °C (200 W). Then the solvent was evaporated, and the residual syrup was purified by column chromatography (1:3 EtOAc/hexane) to give 0.32 g (46%) orange amorphous solid. *R*_f_ = 0.45 (1:1 EtOAc/hexane); [α]_D_ +63 (c 0.52, CHCl_3_); ECD (MeCN) λ_max_ (Δε) 360 (+1.16), 327 (−0.08), 299 (+5.14), 268 (−2.93), 242sh (−0.61), 220 (+2.21), 201 (−7.41) nm; ^1^H-NMR (360 MHz, CDCl_3_) δ (ppm): 8.11 (2H, d, *J* = 7.4 Hz, aromatics), 7.76 (1H, t, *J* = 7.5 Hz, aromatics), 7.57 (2H, t, *J* = 7.8 Hz, aromatics), 6.12 (1H, pseudo t, *J* = 9.6 Hz, H-3’ or H-4’), 5.75 (1H, d, *J* = 9.7 Hz, H-2’), 5.26 (1H, pseudo t, *J* = 9.9 Hz, H-4’ or H-3’), 4.88 (1H, ddd, *J* = 10.3, 4.0, 2.0 Hz, H-5’), 4.29 (1H, dd, *J* = 12.7, 4.1 Hz, H-6’a), 4.09 (1H, dd, *J* = 12.7, 2.0 Hz, H-6’b), 2.08 × 2, 2.01, 1.94 (4 × 3H, 4 s, CH_3_); ^13^C-NMR (90 MHz, CDCl_3_) δ (ppm): 194.7 (C=N), 187.0 (CON), 170.5, 169.6, 169.5, 169.1 (C=O), 136.4, 131.3, 129.3, 129.2 (aromatics), 94.0 (C-1’), 71.2, 70.3, 69.8, 67.4 (C-2’–C-5’), 61.6 (C-6’), 20.7, 20.5 × 2, 20.4 (4 × CH_3_). ESI-MS positive mode (*m*/*z*): calcd for C_22_H_23_NNaO_10_S ([M + Na]^+^): 516.093. Found: 516.095.

*(1’R)-1’,5’-Anhydro-d-glucitol-spiro-[1’,5]-2-phenylthiazolin-4-one* (**16**) *and (2S,1’R)-1’,5’-anhydro-d-glucitol-spiro-[1’,5]-2-methoxy-2-phenylthiazolidin-4-one* (**17**). Compound **15** (100 mg, 0.20 mmol) was dissolved in dry MeOH (10 mL), cooled in an ice-bath and LiOH (2.5 mg, 0.10 mmol) was added. The mixture was stirred until TLC had indicated disappearance of the starting material (3:7 MeOH/CHCl_3_, 7 h). Then the mixture was neutralised with a cation exchange resin Amberlyst 15 (H^+^ form) and the resin was filtered off and the solvent was removed. The purification by column chromatography (1:10 MeOH/CHCl_3_) provided 46 mg yellow syrup as a mixture of **16** (32%) and **17** (32%). Based on ^1^H-NMR spectrum the ratio of thiazolinone to thiazolidinone was ~ 1:1. R_f_ = 0.50 (3:7 MeOH/CHCl_3_); ^1^H-NMR (360 MHz, CD_3_OD) δ (ppm): 8.11 (d, *J* = 7.6 Hz, aromatics), 7.77 (t, *J* = 7.4 Hz, aromatics), 7.72–7.65 (m, aromatics), 7.60 (t, *J* = 7.7 Hz, aromatics), 7.36–7.24 (m, aromatics), 4.44–4.27 (m, 2 × H-3’, 2 × H-5’), 3.95 (d, *J* = 9.3 Hz, H-2’), 3.88–3.80 (m, 2 × H-6’a), 3.74–3.67 (m, 2 × H-6’b), 3.62 (s, OCH_3_), 3.49–3.35 (m, H-2’, 2 × H-4’); ^13^C-NMR (90 MHz, CD_3_OD) δ (ppm): 197.0, 191.0 (thiazolinone C-2, C-4), 173.8 (HNC=O), 144.1–126.6 (aromatics), 99.3, 98.6, 94.0 (thiazolidinone C-1’, C-2, thiazolinone C-1’), 78.7, 77.9, 75.4, 74.9, 74.7, 73.9, 71.2, 70.7 (2 × C-2’–C-5’), 62.9, 62.6 (2 × C-6’), 51.3 (OCH_3_). ESI-MS positive mode (m/z): calcd for compound **16** C_14_H_15_NNaO_6_S ([M + Na]^+^): 348.051, found: 348.052; and calcd for compound **17** C_15_H_19_NNaO_7_S ([M + Na]^+^): 380.077, found: 380.079.

*(2S,1’R)-2’-O-Acetyl-1’,5’-anhydro-d-glucitol-spiro-[1’,5]-2-methoxy-2-phenylthiazolidin-4-one* (**18**). Compound **15** (75 mg, 0.15 mmol) was dissolved in dry MeOH (5 mL) and KHSO_4_ (20 mg, 0.15 mmol) was added. The reaction mixture was kept at r.t. until completion of the transformation (TLC, 1:5 MeOH/CHCl_3_, 14 days). Then the solvent was removed in vacuo and the purification by column chromatography (1:10 MeOH/CHCl_3_) gave 15 mg yellow syrup of **18** (25%). *R*_f_ = 0.30 (1:5 MeOH/CHCl_3_); [α]_D_ +50 (c 0.75, MeOH); ^1^H-NMR (360 MHz, CD_3_OD) δ (ppm): 7.57–7.51 (2H, m, aromatics), 7.39–7.32 (3H, m, aromatics), 5.01 (1H, d, *J* = 9.6 Hz, H-2’), 4.54 (1H, pseudo t, *J* = 9.3 Hz, H-3’ or H-4’), 4.28 (1H, ddd, *J* = 9.9, 5.0, 2.0 Hz, H-5’), 3.86 (1H, dd, *J* = 12.1, 2.0 Hz, H-6’a), 3.72 (1H, dd, *J* = 12.0, 5.0 Hz, H-6’b), 3.61 (3H, s, OCH_3_), 3.46 (1H, pseudo t, *J* = 9.5 Hz, H-4’ or H-3’), 2.00 (3H, s, CH_3_); ^13^C-NMR (90 MHz, CD_3_OD) δ (ppm): 172.8, 171.3 (C=O), 144.1, 129.8, 129.4, 126.2 (aromatics), 98.6, 92.1 (C-1’, C-2), 79.1, 74.5, 73.2, 71.1 (C-2’–C-5’), 62.8 (C-6’), 51.6 (OCH_3_), 21.1 (CH_3_). ESI-MS positive mode (*m*/*z*): calcd for C_17_H_21_NNaO_8_S ([M + Na]^+^): 422.088. Found: 422.088.

*(2S,1’R)-1’,5’-Anhydro-2’,3’,4’,6’-tetra-O-benzoyl-d-glucitol-spiro-[1’,5]-2-methoxy-2-phenylthiazolidin-4-one* (**19**). Prepared from **2** (30 mg, 0.04 mmol) in MeOH according to general procedure II. Orange syrup, *R*_f_ = 0.38 (1:1:3 EtOAc/hexane/toluene); ^1^H-NMR (360 MHz, CDCl_3_) δ (ppm): 8.06–7.95 (6H, m, aromatics), 7.81 (2H, d, *J* = 7.6 Hz, aromatics), 7.63–7.23 (14H, m, aromatics), 7.03 (1H, t, *J* = 7.5 Hz, aromatics), 6.87 (1H, pseudo t, *J* = 9.7 Hz, H-3’ or H-4’), 6.72 (2H, t, *J* = 7.8 Hz, aromatics), 6.65 (1H, s, NH), 5.89 (1H, d, *J* = 9.7 Hz, H-2’), 5.73 (1H, pseudo t, *J* = 10.0 Hz, H-4’ or H-3’), 5.19 (1H, ddd, *J* = 10.2, 5.8, 2.9 Hz, H-5’), 4.60 (1H, dd, *J* = 12.2, 2.9 Hz, H-6’a), 4.53 (1H, dd, *J* = 12.2, 5.8 Hz, H-6’b), 3.53 (3H, s, OCH_3_); ^13^C-NMR (100 MHz, CDCl_3_) δ (ppm): 170.4 (C-4), 166.1, 165.6, 165.5, 164.7 (C=O), 140.6 (q, Ph), 133.6–125.5 (aromatics), 97.2, 90.5 (C-1’, C-2), 73.3, 71.4, 71.1, 69.4 (C-2’–C-5’), 63.5 (C-6’), 51.3 (OCH_3_). ESI-MS positive mode (*m*/*z*): calcd for C_43_H_35_NNaO_11_S ([M + Na]^+^): 796.182. Found: 796.180.

The following experiment indicated the reversibility of the methanol addition: Compound **19** was dissolved in dry toluene (10 mL/mmol) and heated at 80 °C. After 3 h TLC (1:1:3 EtOAc/hexane/toluene) indicated complete transformation to give, after removal of the solvent, compound **2** in a quantitative yield.

*(2S,1’R)-1’,5’-Anhydro-2’,3’,4’,6’-tetra-O-benzoyl-d-glucitol-spiro-[1’,5]-2-ethoxy-2-phenylthiazolidin-4-one* (**20**). Prepared from **2** (65 mg, 0.09 mmol) in MeOH according to general procedure II. Orange syrup, *R*_f_ = 0.40 (1:1:3 EtOAc/hexane/toluene); ^1^H-NMR (400 MHz, CDCl_3_) δ (ppm): 8.08–7.95 (6H, m, aromatics), 7.80 (2H, d, *J* = 7.6 Hz, aromatics), 7.64–7.22 (14H, m, aromatics), 7.01 (1H, t, *J* = 7.2 Hz, aromatics), 6.95 (1H, br s, NH), 6.88 (1H, pseudo t, *J* = 9.7 Hz, H-3’ or H-4’), 6.71 (2H, t, *J* = 7.9 Hz, aromatics), 5.89 (1H, d, *J* = 9.7 Hz, H-2’), 5.72 (1H, pseudo t, *J* = 9.9 Hz, H-4’ or H-3’), 5.23 (1H, ddd, *J* = 10.0, 5.4, 2.9 Hz, H-5’), 4.55 (2H, m, H-6’a, H-6’b), 4.18–4.07 (1H, m, OC*H*_2_CH_3_), 3.67–3.62 (1H, m, OC*H*_2_CH_3_), 1.12 (3H, t, *J* = 7.0 Hz, OCH_2_C*H*_3_); ^13^C-NMR (100 MHz, CDCl_3_) δ (ppm): 170.3 (C-4), 166.1, 165.5, 165.5, 164.7 (C=O), 140.9 (q, Ph), 133.6–125.4 (aromatics), 96.3, 90.5 (C-1’, C-2), 73.3, 71.4, 71.1, 69.4 (C-2’–C-5’), 63.7 (C-6’), 59.7 (O*C*H_2_CH_3_), 14.5 (OCH_2_*C*H_3_). ESI-MS positive mode (*m*/*z*): calcd for C_44_H_37_NNaO_11_S ([M + Na]^+^): 810.198. Found: 810.195.

*(2S,1’R)-2’,3’,4’,6’-Tetra-O-acetyl-1’,5’-anhydro-d-glucitol-spiro-[1’,5]-2-methoxy-2-phenylthiazolidin-4-one* (**21**). Prepared from **15** (85 mg, 0.17 mmol) in MeOH according to general procedure II. Orange syrup; *R*_f_ = 0.28 (1:2 EtOAc/toluene); ECD (MeCN) λ_max_ (Δε) 302 (+0.29), 267sh (−0.84), 248 (−2.57), 219 (−2.52), 209sh (−1.29), 203sh (+0.45) nm; ^1^H-NMR (400 MHz, CDCl_3_) δ (ppm): 7.51 (2H, dd, *J* = 6.7, 2.9 Hz, aromatics), 7.35–7.29 (3H, m, aromatics), 7.12 (1H, br s, NH), 6.09 (1H, pseudo t, *J* = 9.6 Hz, H-3’ or H-4’), 5.27 (1H, d, *J* = 9.6 Hz, H-2’), 5.13 (1H, pseudo t, *J* = 9.9 Hz, H-4’ or H-3’), 4.68 (1H, ddd, *J* = 10.2, 4.8, 2.2 Hz, H-5’), 4.19 (1H, dd, *J* = 12.4, 5.0 Hz, H-6’a), 4.09 (1H, dd, *J* = 12.4, 2.2 Hz, H-6’b), 3.56 (3H, s, OCH_3_), 2.04, 2.02, 1.97, 1.95 (4 × 3H, 4 s, 4 × CH_3_); ^13^C-NMR (100 MHz, CDCl_3_) δ (ppm): 170.8, 170.3, 170.0, 169.7, 169.2 (C=O, C-4), 141.5, 129.2, 125.5, 124.9 (aromatics), 97.3, 90.1 (C-1’, C-2), 72.5, 71.2, 70.3, 68.0 (C-2’–C-5’), 62.3 (C-6’), 51.1 (OCH_3_), 20.6 (3), 20.5 (4 × CH_3_). ESI-MS positive mode (*m*/*z*): calcd for C_23_H_27_NNaO_11_S ([M + Na]^+^): 548.120. Found: 548.120.

The following experiment indicated the reversibility of the methanol addition: Compound **21** was dissolved in dry toluene (10 mL/mmol) and heated at 60 °C. After 2 h TLC (1:2 EtOAc/toluene) indicated complete transformation to give, after removal of the solvent, compound **15** in a quantitative yield.

*(2S,1’R)-2’,3’,4’,6’-Tetra-*O*-acetyl-1’,5’-anhydro-d-glucitol-spiro-[1’,5]-2-ethoxy-2-phenylthiazolidin-4-one* (**22**). Prepared from **15** (30 mg, 0.06 mmol) in MeOH according to general procedure II. Orange syrup; *R*_f_ = 0.26 (1:3 EtOAc/toluene); ^1^H-NMR (400 MHz, CDCl_3_) δ (ppm): 7.54 (2H, dd, *J* = 6.8, 2.9 Hz, aromatics), 7.36–7.31 (3H, m, aromatics), 6.79 (1H, br s, NH), 6.10 (1H, pseudo t, *J* = 9.5 Hz, H-3’ or H-4’), 5.28 (1H, d, *J* = 9.7 Hz, H-2’), 5.13 (1H, pseudo t, *J* = 9.9 Hz, H-4’ or H-3’), 4.70 (1H, ddd, *J* = 10.3, 5.0, 2.2 Hz, H-5’), 4.19 (1H, dd, *J* = 12.4, 5.1 Hz, H-6’a), 4.11 (1H, dd, *J* = 12.4, 2.2 Hz, H-6’b), 4.10–4.08 (1H, m, OC*H*_2_CH_3_), 3.69–3.64 (1H, m, OC*H*_2_CH_3_), 2.07, 2.04, 1.99, 1.96 (4 × 3H, 4 s, CH_3_), 1.31 (3H, t, *J* = 7.0 Hz, OCH_2_C*H*_3_); ^13^C-NMR (100 MHz, CDCl_3_) δ (ppm): 170.7, 170.0, 169.9, 169.7, 169.1 (C=O, C-4), 141.7, 129.4, 128.7, 125.1 (aromatics), 96.2, 90.0 (C-1’, C-2), 72.8, 71.3, 70.4, 68.1 (C-2’–C-5’), 62.5 (C-6’), 59.9 (O*C*H_2_CH_3_), 20.8, 20.7 (3) (4 × CH_3_), 14.9 (OCH_2_*C*H_3_). ESI-MS positive mode (*m*/*z*): calcd for C_24_H_29_NNaO_11_S ([M + Na]^+^): 562.135. Found: 562.134.

*(1’R)-2’,3’,4’,6’-Tetra-O-acetyl-1’,5’-anhydro-d-glucitol-spiro-[1’,5]-2-phenylthiazolin-4-one (15) and (1’R)-2’,3’,4’,6’-tetra-O-acetyl-1’,5’-anhydro-d-glucitol-spiro-[1’,5]-2-hydroxy-2-phenylthiazolidin-4-one* (**23**). Water (0.1 mL) was added to the solution of spiro thiazolinone **15** (50 mg, 0.10 mmol) in toluene (3 mL) and the mixture was stirred for 6 days at rt, then the solvent was evaporated. Based on ^1^H-NMR spectrum the ratio of thiazolinone to thiazolidinone was ~1:0.4. Yellow syrup, R_f_ = 0.45 (1:1 EtOAc/hexane); ^1^H-NMR (400 MHz, CDCl_3_) δ (ppm): 8.11 (d, *J* = 7.4 Hz, aromatics), 7.76 (t, *J* = 7.5 Hz, aromatics), 7.58–7.53 (m, aromatics), 7.36–7.32 (m, aromatics), 6.82 (s, NH), 6.12 (2 × pseudo t, *J* = 9.6 Hz, 2 × H-3’), 5.74 (d, *J* = 9.7 Hz, thiazolinone H-2’), 5.30 (d, *J* = 9.7 Hz, thiazolidinone H-2’), 5.25 (pseudo t, *J* = 9.9 Hz, thiazolinone H-4’), 5.15 (pseudo t, *J* = 9.9 Hz, thiazolidinone H-4’), 4.87 (ddd, *J* = 10.3, 4.1, 2.1 Hz, thiazolinone H-5’), 4.73 (ddd, *J* = 10.3, 5.1, 2.2 Hz, thiazolidinone H-5’), 4.27 (dd, *J* = 12.8, 4.2 Hz, thiazolinone H-6’a), 4.21 (dd, *J* = 12.4, 5.1 Hz, thiazolidinone H-6’a), 4.13 (dd, *J* = 12.4, 2.2 Hz, thiazolidinone H-6’b), 4.08 (dd, *J* = 12.7, 2.0 Hz, thiazolinone H-6’b), 2.08, 2.08, 2.07, 2.05, 2.00, 2.00, 1.97, 1.93 (8 s, 8 × CH_3_). ESI-MS positive mode (m/z): calcd for compound **15** C_22_H_23_NNaO_10_S ([M + Na]^+^): 516.093, found: 516.091; and calcd for compound **23** C_22_H_25_NNaO_11_S ([M + Na]^+^): 534.104, found: 534.103.

### 4.3. Computational Methods

Mixed torsional/low-frequency mode conformational searches were carried out by means of the Macromodel 10.8.011 software [39] using OPLS-2005 (Optimized Potential for Liquid Simulations) Force Field [40,41] with the implicit solvent model for CHCl_3_ applying a 21 kJ/mol energy window yielding 53–151 conformers. Geometry optimizations [B3LYP/6-31G(d) in vacuo and ωB97XD/TZVP [35,38] with a PCM solvent model for MeCN] and TDDFT calculations were performed with Gaussian 09 using various functionals (B3LYP, BH&HLYP, CAM-B3LYP and PBE0) and TZVP basis set [42]. ECD spectra were generated as the sum of Gaussians with 3000 cm^−1^ half-height width (corresponding to ca. 15 nm at 220 nm), using dipole-velocity computed rotational strengths [43]. Boltzmann distributions were estimated from the ZPVE-corrected B3LYP/6-31G(d) energies in the gas-phase calculations and from the ωB97XD/TZVP energies in the solvated ones. The MOLEKEL software package was used for visualization of the results [44].

## Figures and Tables

**Table 1:** Synthesis of spiro-thiazolinones **2**–**5**.

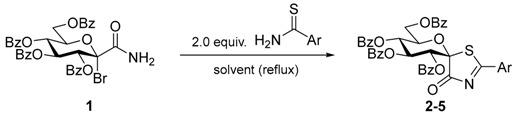					
The observed by-products:		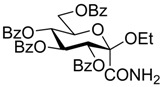	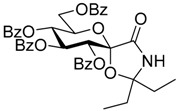	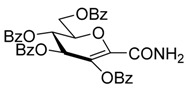	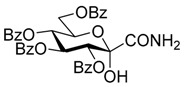
		6 [25]	7 [26]	8 [27]	9 [28]
Entry	Ar	Solvent	Reaction Time	Product	Yield ^1^ (%)
1.	Ph	Ethanol	18 h	**6**	65
2.	Ph	Pentan-3-one	3 days	**7**	33
3.	Ph	Pyridine	0.5 h	**8**	22
4.	Ph	DMF	2 h	**9**	75
5.	Ph	Toluene	1.5 days	**2**	34
6.	Ph	*m*-Xylene	4 h	**2**	33
7.	Ph	Dibutyl ether	1 days	**2**	26
8.	Ph	Anisole ^2^	1 days	**2**	27
9.	Ph	Dioxane	1.5 days	**2**	28
10.	Ph	Nitromethane	1.5 days	**2**	33
11.	Ph	*m*-Xylene/Ar atm.	4 h	**2**	27
12.	Ph	*m*-Xylene ^3^	2.5 h	**2**	30
13.	Ph	*m*-Xylene (MW: 120 °C, 200 W)	1 h	**2**	40
14.	Ph	*m*-Xylene (MW: 140 °C, 200 W)	1.5 h	**2**	53
15.	Ph	*m*-Xylene (MW: 140 °C, 200 W) ^4^	1.5 h	**2**	13
16.	1-Napth	*m*-Xylene (MW: 140 °C, 200 W)	1.5 h	**3**	32
17.	2-Napth	*m*-Xylene (MW: 140 °C, 200 W)	1.5 h	**4**	40
18.	4-Me-Ph	*m*-Xylene (MW: 140 °C, 200 W)	1.5 h	**5**	53

^1^ Isolated yield; ^2^ Oil bath temperature was 130 °C; ^3^ With added AgOTf (1.1 equiv.); ^4^ With 1.1 equiv. of thiobenzamide.

**Table 2:** Observations during experiments for deacetylation [29] of spiro-thiazolinone **15**.

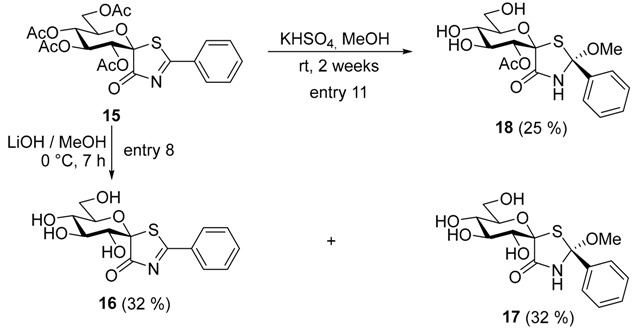		
Entry	Conditions	Observations
1.	cat. NaOMe/MeOH, 1 day	Complex reaction mixture
2.	K_2_CO_3_, CHCl_3_: MeOH = 6:1, 1 day	Decomposition
3.	1 equiv. KCN/MeOH, r.t., 4 weeks	Complex reaction mixture
4.	50% NH_3_/MeOH, 2 h	Complex reaction mixture
5.	NaOH, Bu_4_NHSO_4_/DCM, r.t., 1 day	Decomposition
6.	4 equiv. DBU/Toluene, 60 °C, Ar atm.	Complex reaction mixture
7.	4 equiv. LiOH/MeOH, 0 °C, 1 h	**16** (17%) + **17** (17%)
8.	0.5 equiv. LiOH/MeOH, 0 °C, 7 h	**16** (32%) + **17** (32%)
9.	cat. AcCl/MeOH, r.t., 1 week	Complex reaction mixture
10.	4 Å Molecular sieves/MeOH, r.t., 4 weeks	No reaction
11.	KHSO_4_/MeOH, r.t., 2 weeks	**18** (25%)

**Table 3:** Addition of alcohols and water onto spiro-thiazolinones **2** and **15**.

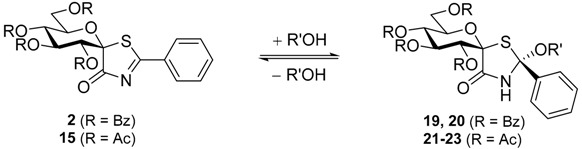			
Entry	Starting Compound	R’	Product ^1^
1.	**2**	Me	**19**
2.		Et	**20**
3.	**15**	Me	**21**
4.		Et	**22**
5.		H	**23** ^2^

^1^ The formation of the other possible diastereomers cannot be excluded although their isolation failed; ^2^ Partial addition (**15**:**23**~1:0.4).

## Supplementary Materials

The following are available online. Copies of ^1^H and ^13^C spectra of new compounds; depictions of low energy conformers of (1’*R*)-**15**, (1’*S*)-**15**, (2*R*,1’*R*)-**21**, (2*S*,1’*R*)-**21** (Figures S1–S4).
