# Supplementary material for: Glucopyranosylidene-Spiro-Thiazolinones: Synthetic Studies and Determination of Absolute Configuration by TDDFT-ECD Calculations
Source: Molecules. 2017 Oct 19;22(10):1760. doi: 10.3390/molecules22101760 (PMC6151563; doi:10.3390/molecules22101760)
Supplement: Supplementary file 1 [file molecules-22-01760-s001.pdf]

Supporting Information

Glucopyranosylidene-spiro-thiazolinones: synthetic studies and determination of absolute configuration by TDDFT-ECD calculations

Katalin E. Szabó, Sándor Kun, Attila Mándi, Tibor Kurtán, László Somsák*

Department of Organic Chemistry, University of Debrecen, H-4002 POB 400, Debrecen, Hungary

Contents

Copies of NMR spectra of the prepared compounds S2

Low energy conformers of (1'*R*)-**15**, (1'*S*)-**15**, (2*R*,1'*R*)-**21**, (2*S*,1'*R*)-**21** S16

* Corresponding author – tel: +3652512400 ext 22348; fax: +3652512744; e-mail: somsak.laszlo@science.unideb.hu

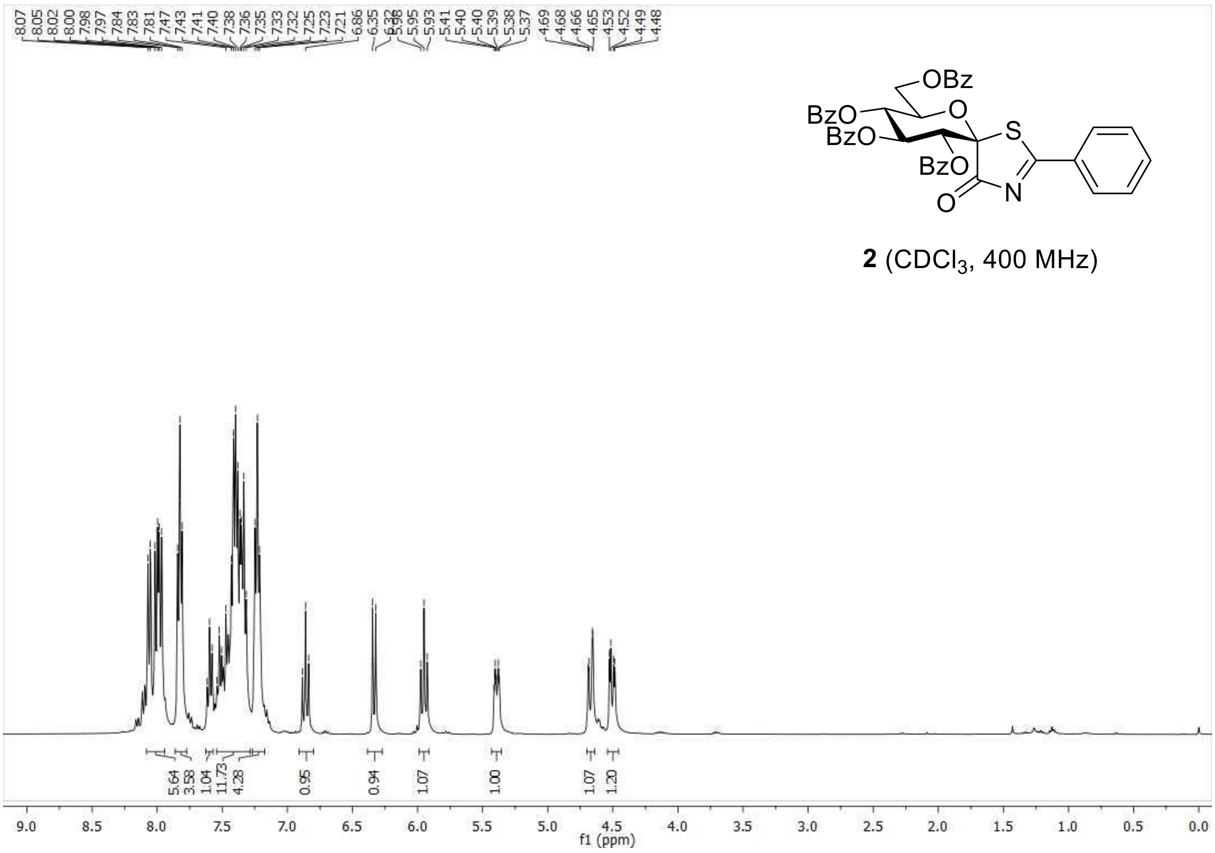

2 ($CDCl_3$, 400 MHz)

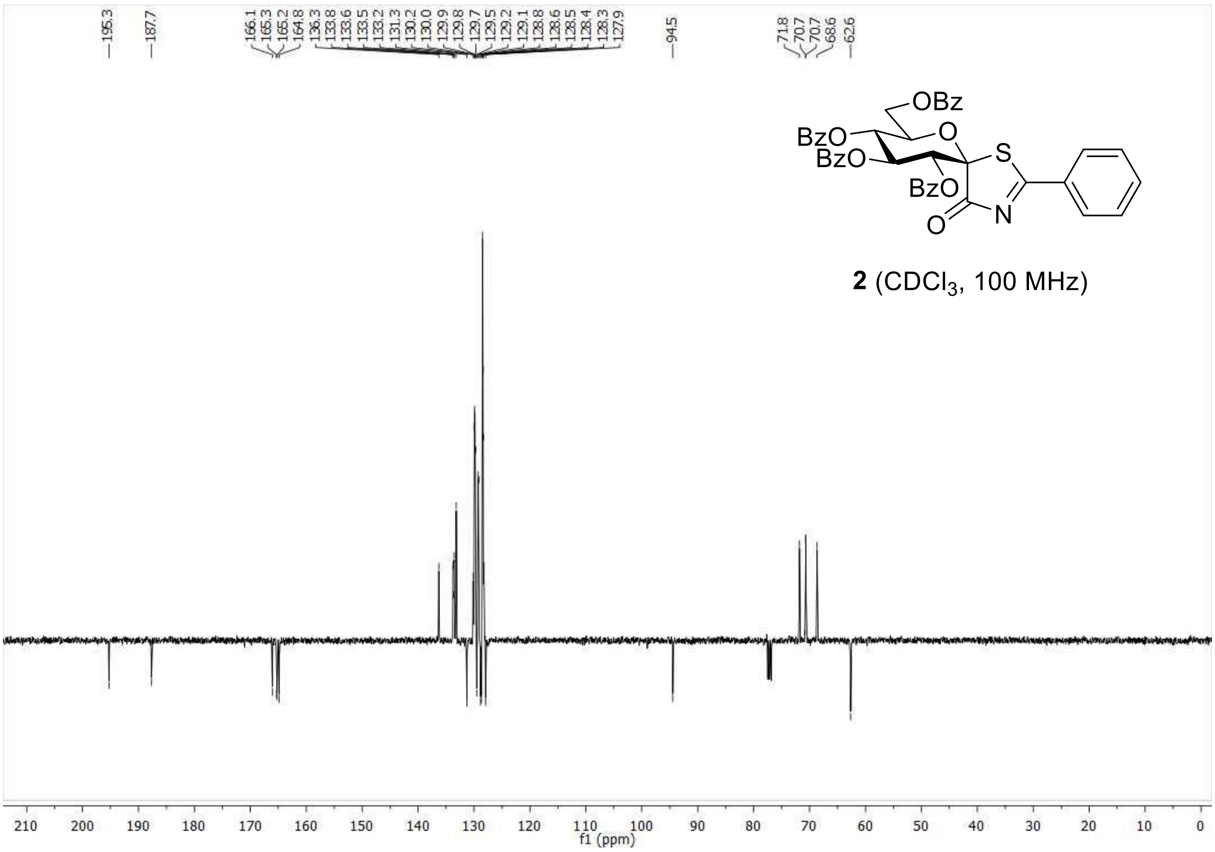

2 ($CDCl_3$, 100 MHz)

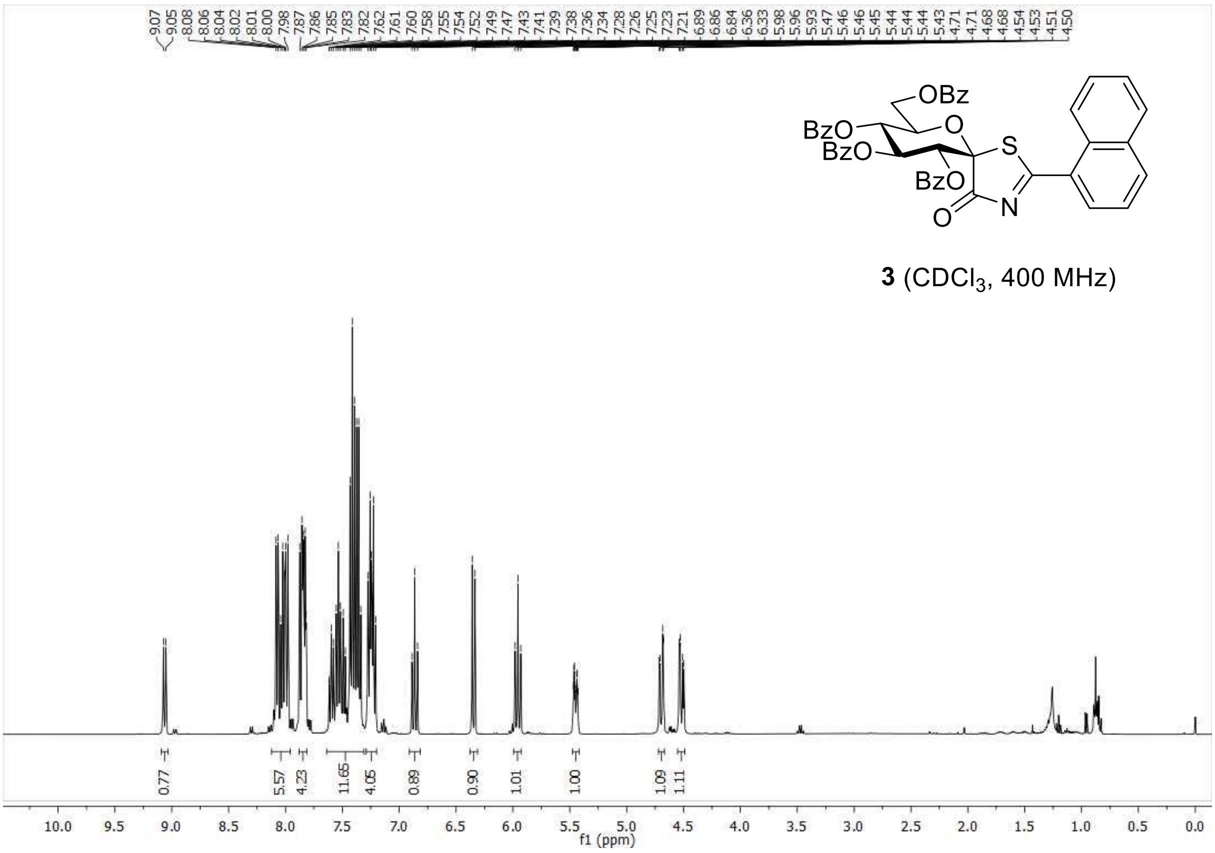
OBz
BzO
BzO
BzO
O
S
N
O
3 (CDCl$_3$, 400 MHz)
f1 (ppm)

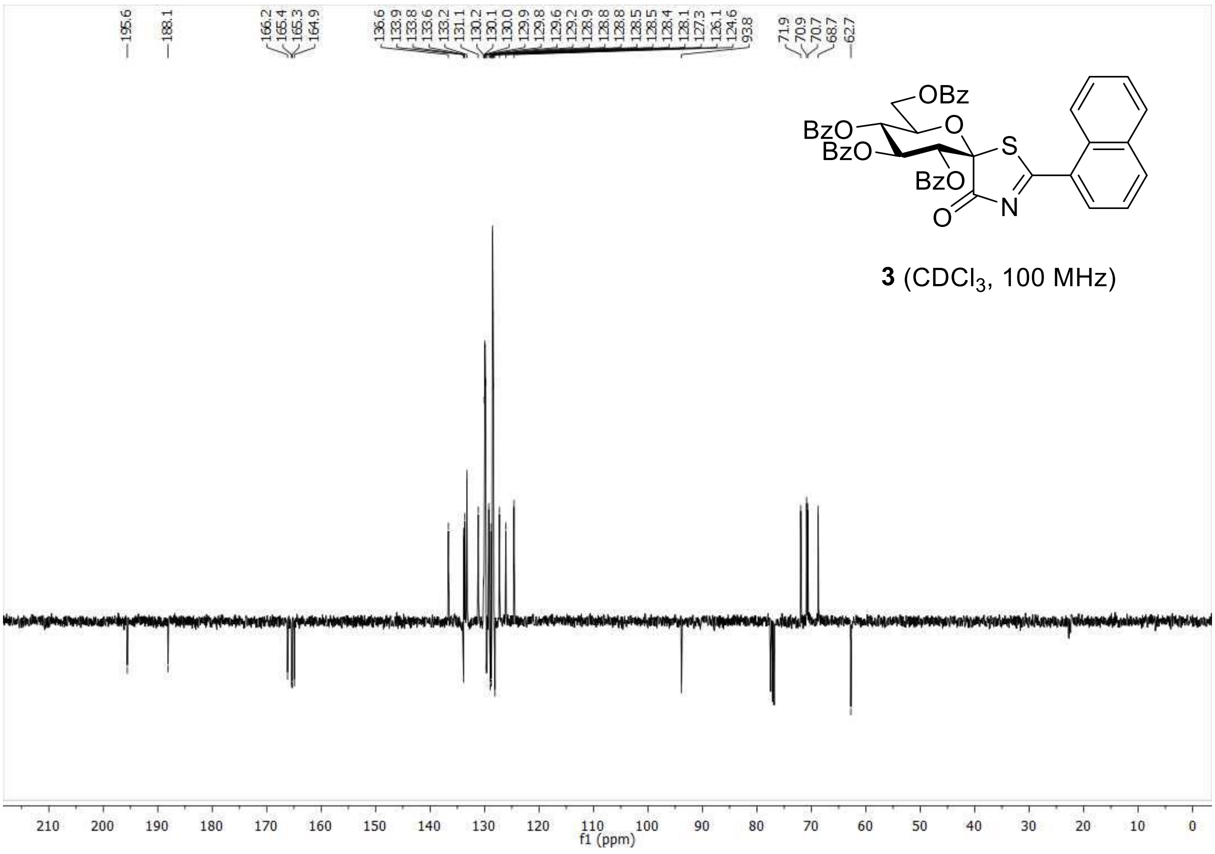
OBz
BzO
BzO
BzO
O
S
N
O
3 (CDCl$_3$, 100 MHz)
f1 (ppm)

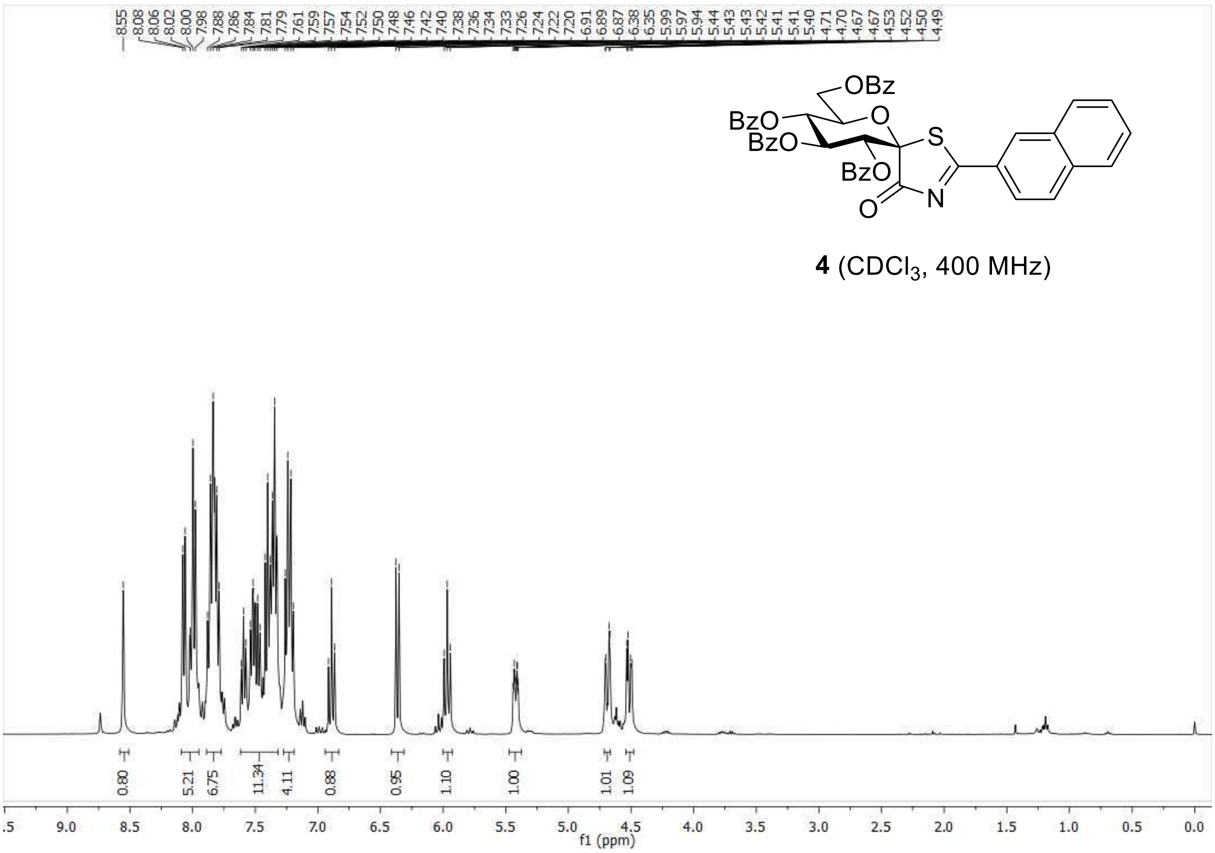

4 ($CDCl_3$, 400 MHz)

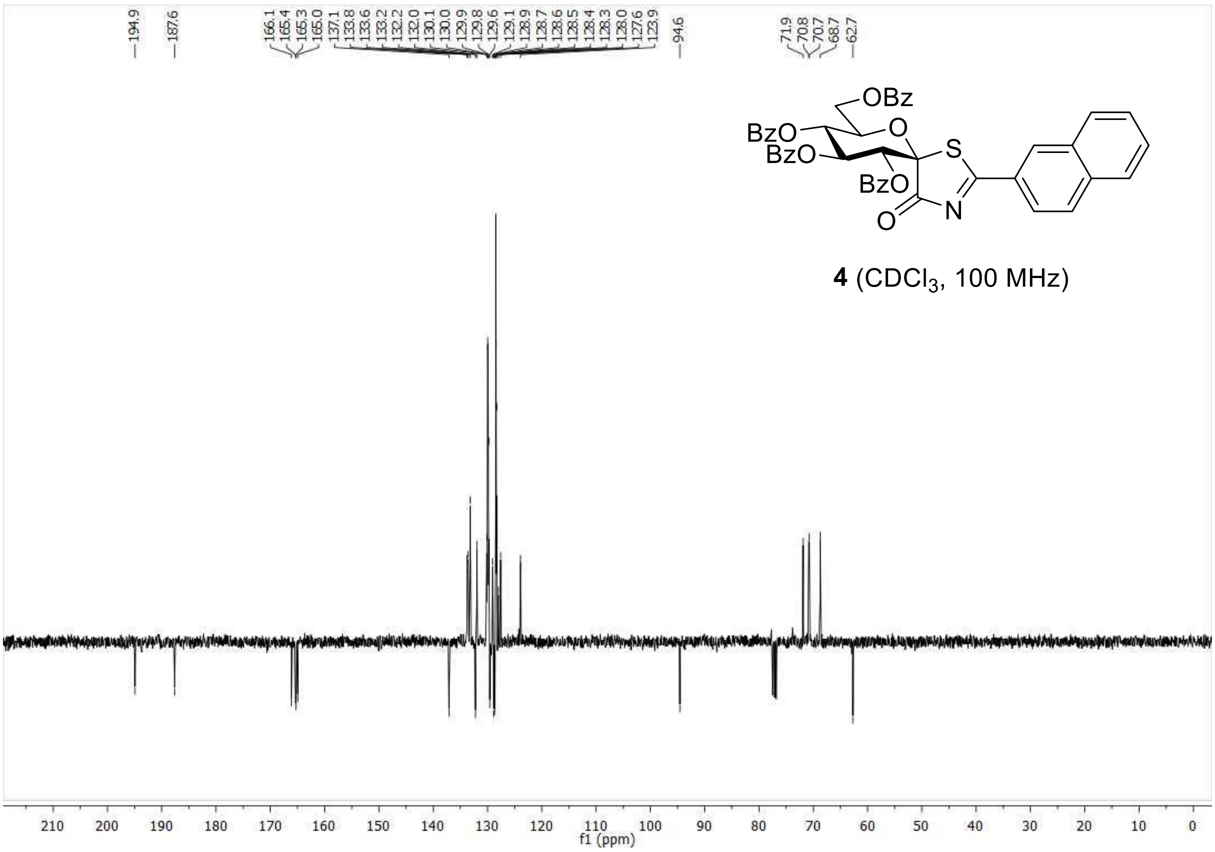

4 ($CDCl_3$, 100 MHz)

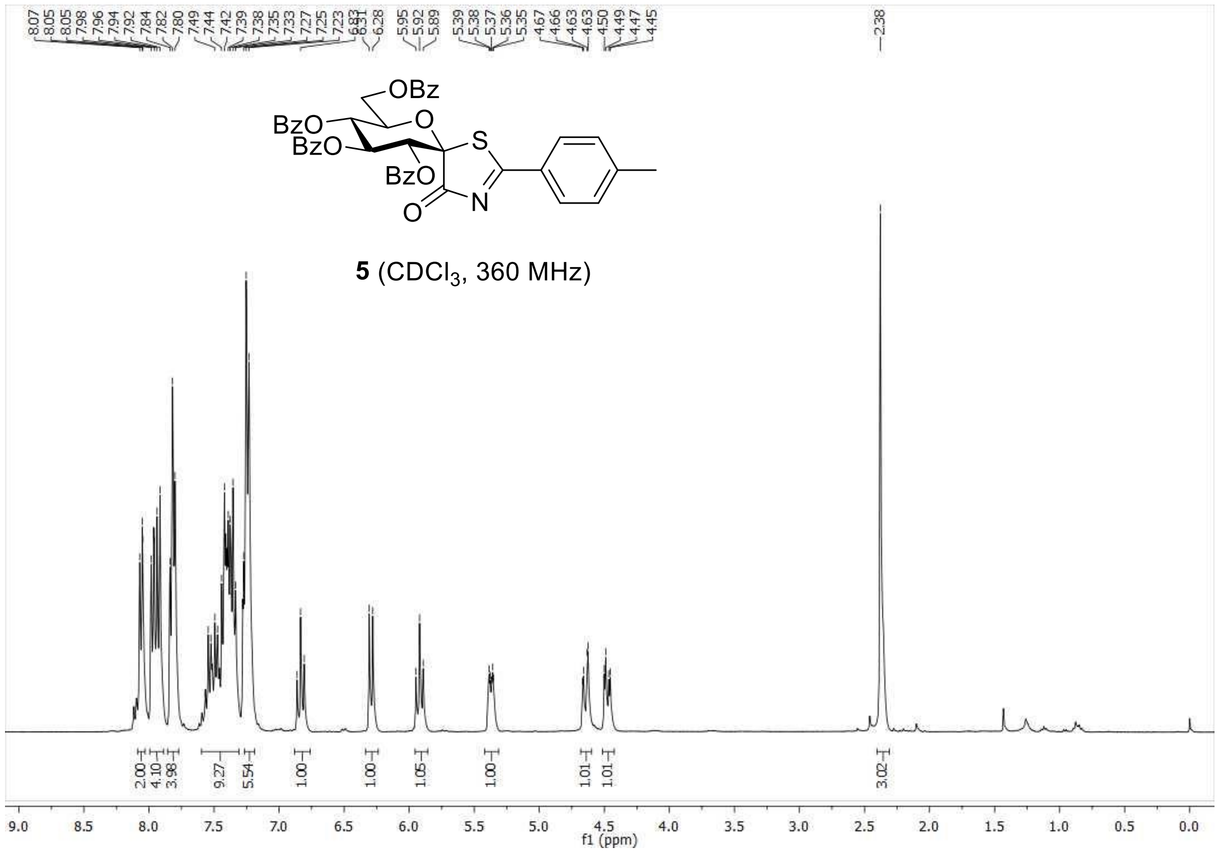

OBz
BzO
BzO
BzO
S
N
O
5 (CDCl3, 360 MHz)
f1 (ppm)

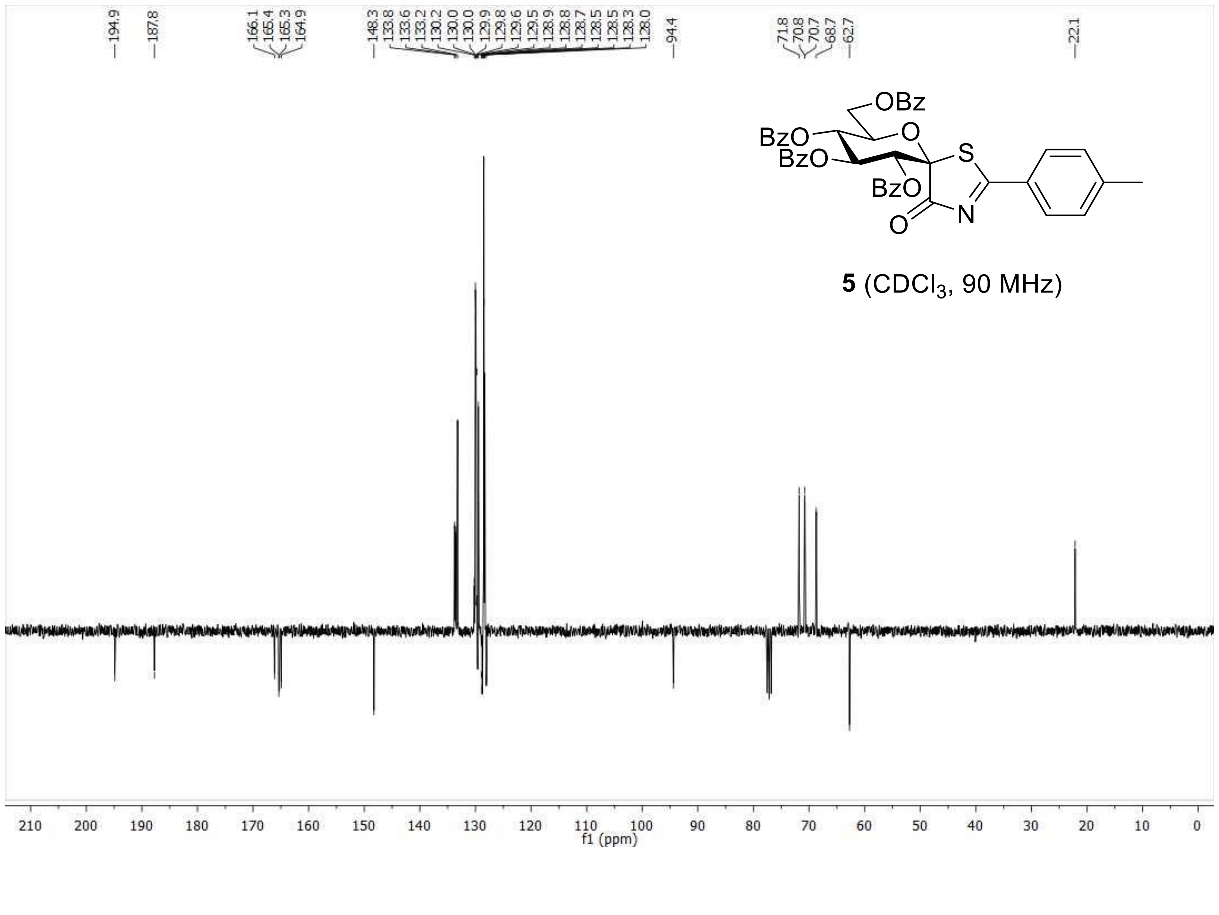

OBz
BzO
BzO
BzO
S
N
O
5 (CDCl3, 90 MHz)
f1 (ppm)

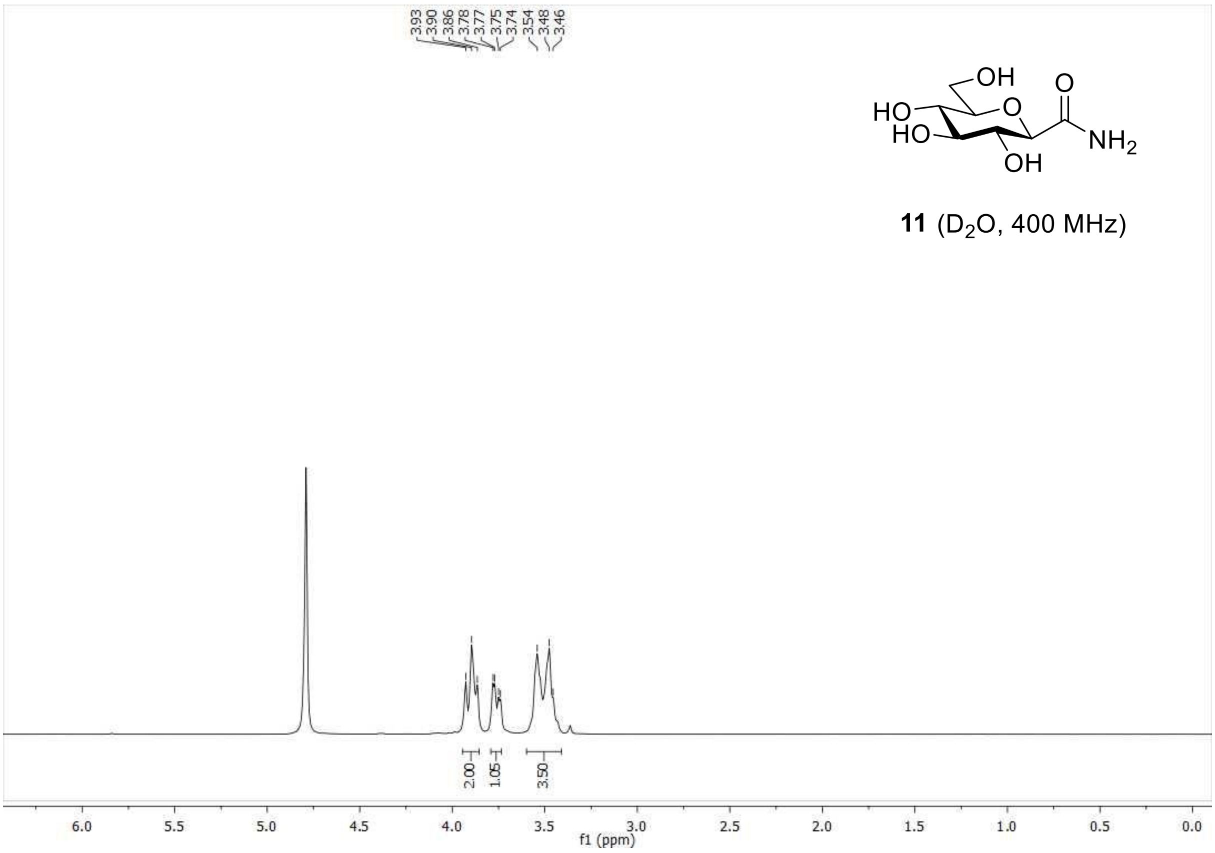

11 (D_2O, 400 MHz)

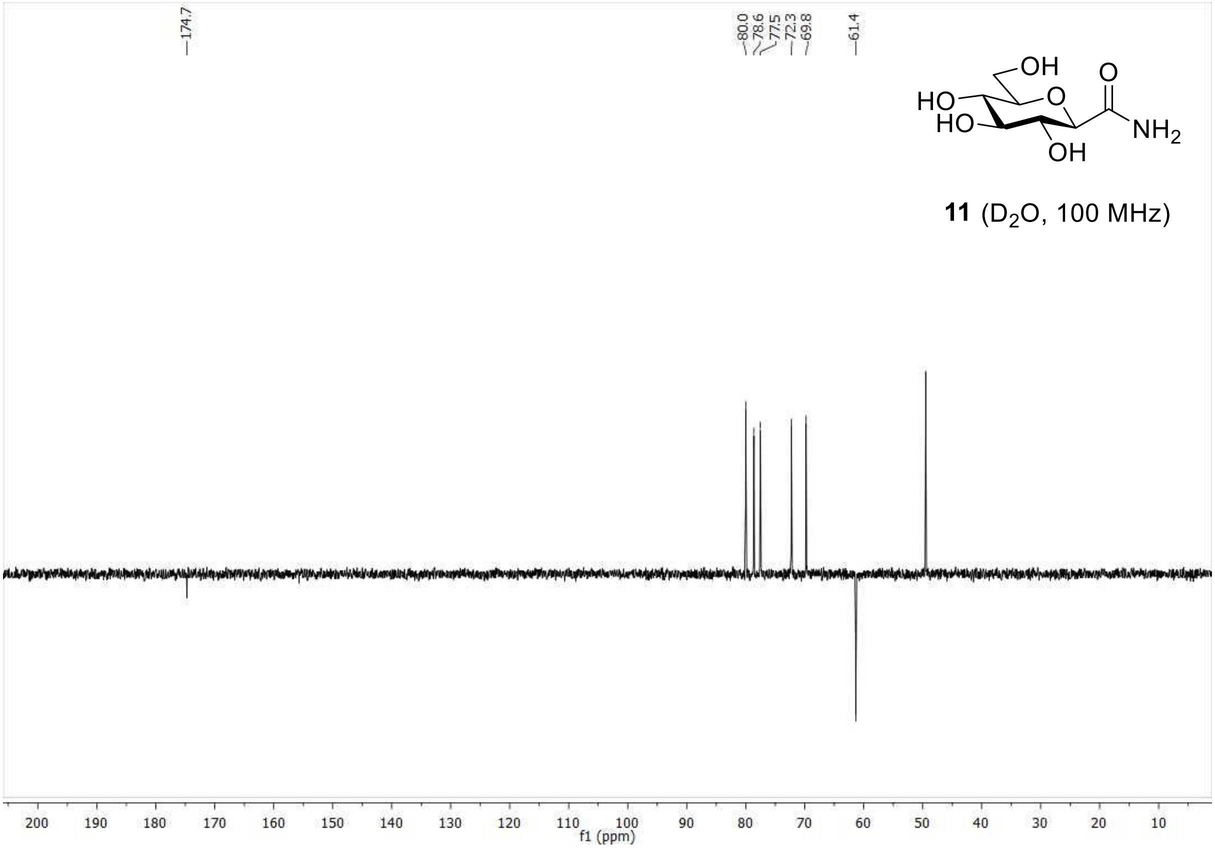

11 (D_2O, 100 MHz)

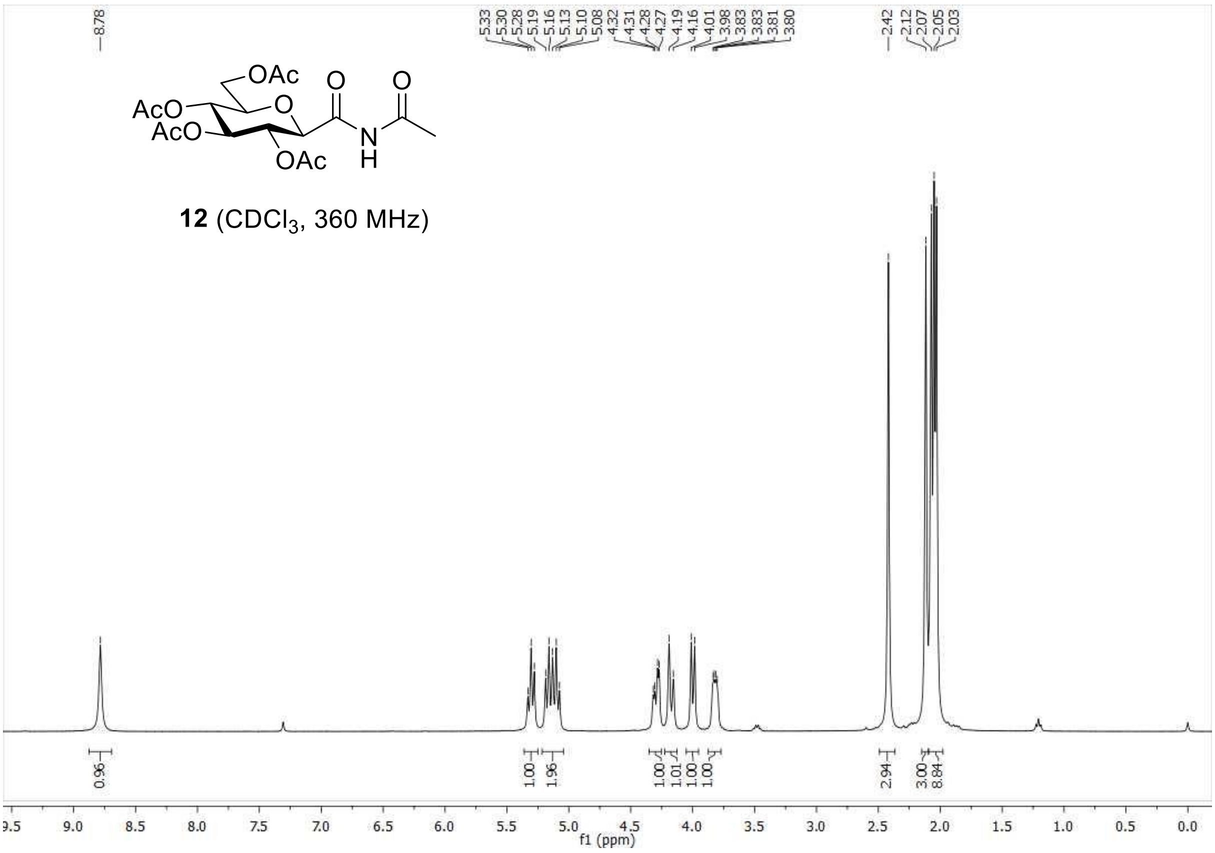

8.78
5.33 5.30 5.28 5.19 5.16 5.13 5.10 5.08 4.32 4.31 4.28 4.27 4.19 4.16 4.01 3.98 3.83 3.83 3.81 3.80
2.42 2.12 2.07 2.05 2.03
OAc
AcO
AcO
O
OAc
O
O
N
H
12 (CDCl3, 360 MHz)
0.96
1.00
1.96
1.00
1.01
1.00
1.00
2.94
3.00
8.84
f1 (ppm)

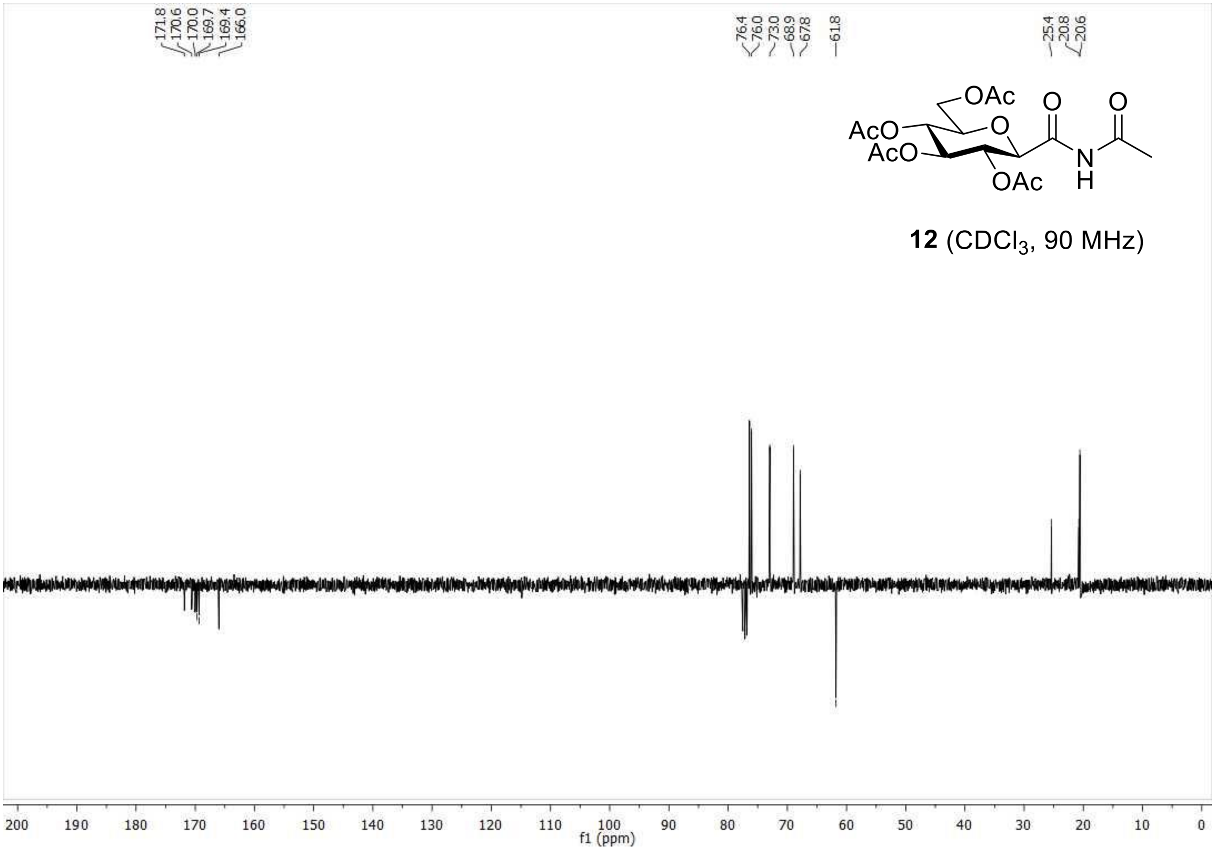

171.8 170.6 170.0 169.7 169.4 166.0
76.4 76.0 73.0 68.9 67.8
61.8
25.4 20.8 20.6
OAc
AcO
AcO
O
OAc
O
O
N
H
12 (CDCl3, 90 MHz)
f1 (ppm)

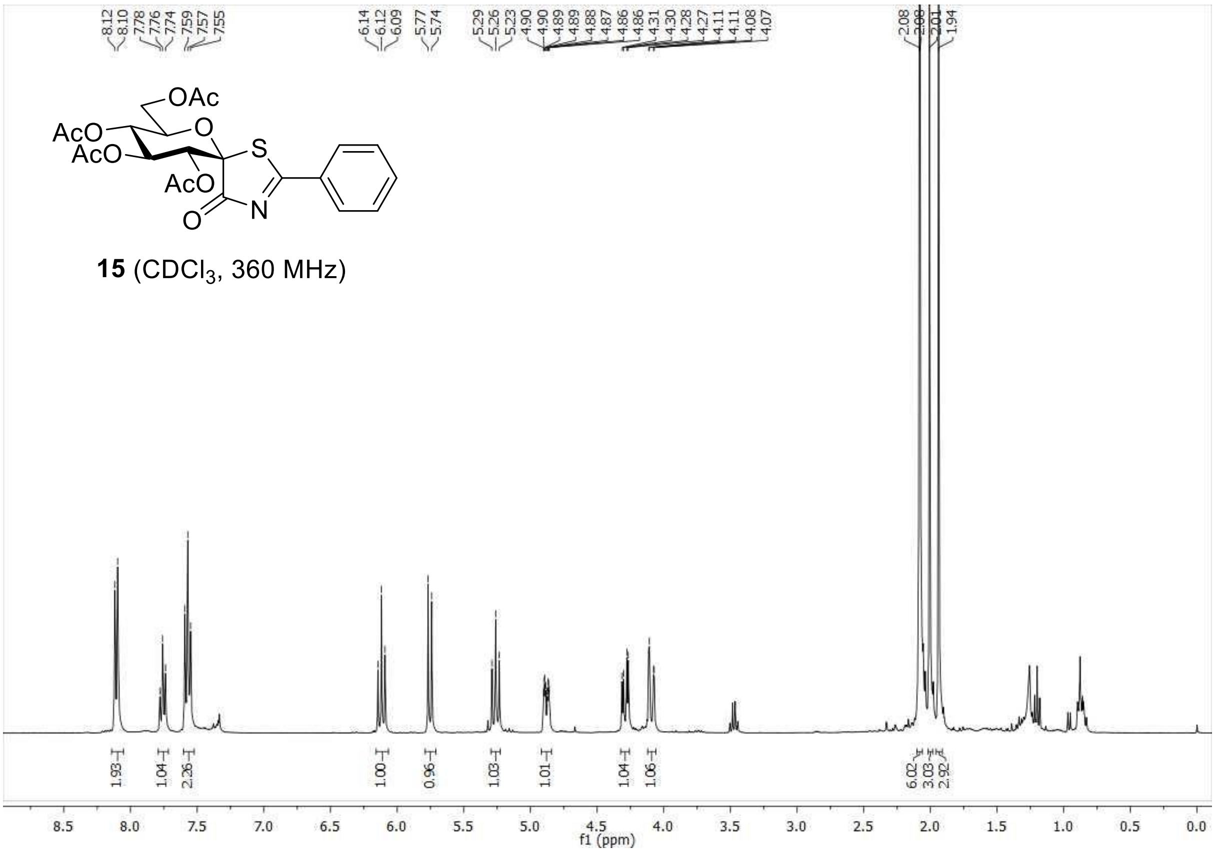
OAc
AcO
AcO
AcO
O
S
N
O
15 (CDCl3, 360 MHz)
f1 (ppm)

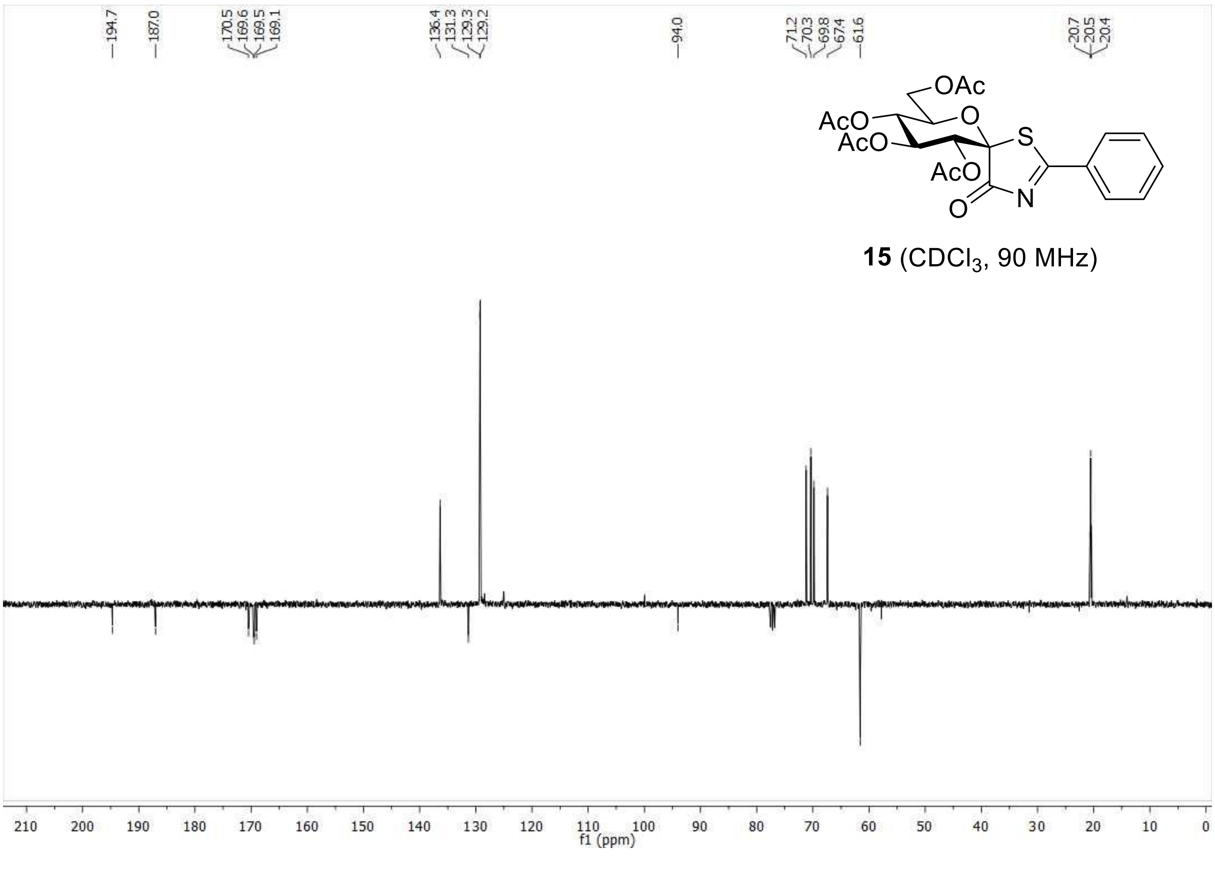
OAc
AcO
AcO
AcO
O
S
N
O
15 (CDCl3, 90 MHz)
f1 (ppm)

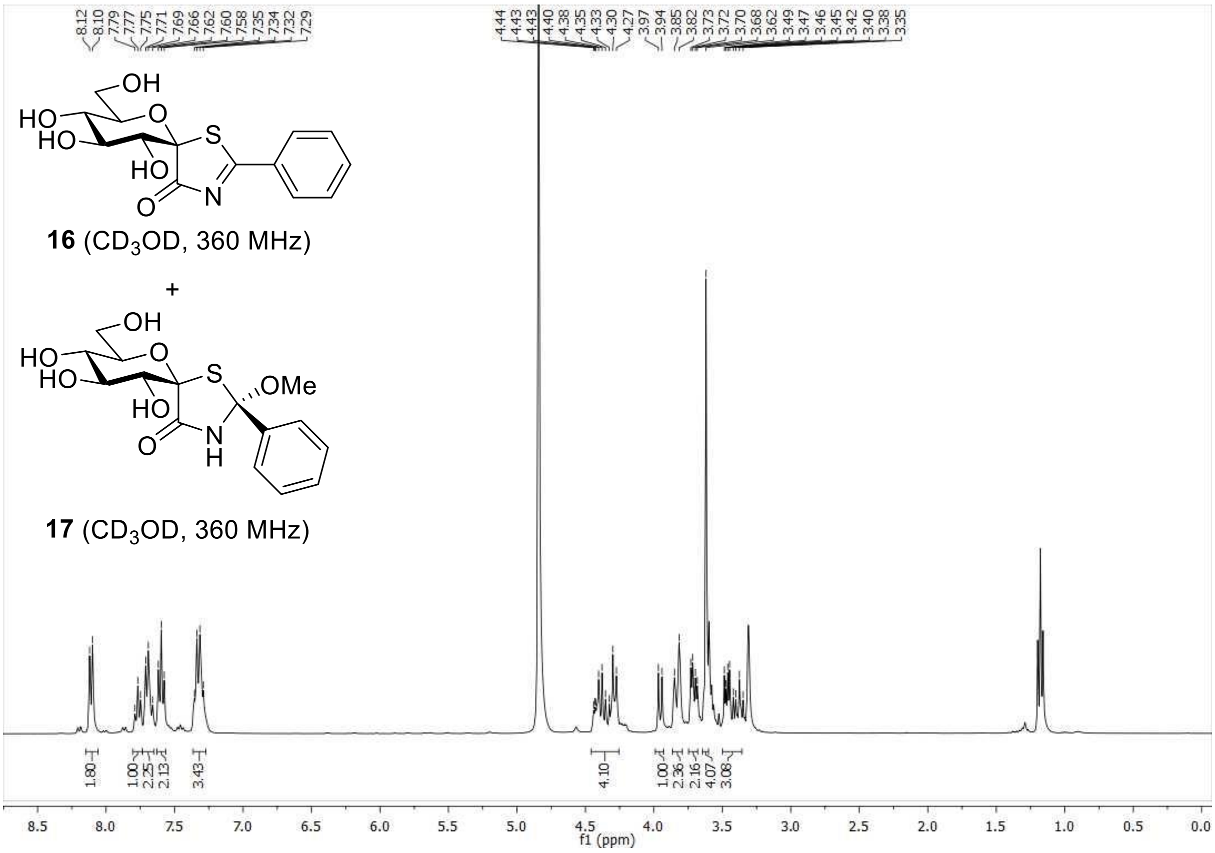

8.12 8.10 7.79 7.77 7.75 7.71 7.69 7.66 7.62 7.60 7.58 7.35 7.34 7.32 7.29
4.44 4.43 4.43 4.40 4.38 4.35 4.33 4.30 4.27 3.97 3.94 3.85 3.82 3.73 3.72 3.70 3.68 3.62 3.49 3.47 3.46 3.45 3.42 3.40 3.38 3.35
OH
HO
HO
HO
O
S
N
O
16 (CD3OD, 360 MHz)
+
OMe
N
H
17 (CD3OD, 360 MHz)
1.80 1.00 2.25 2.13 3.43
4.10
1.00 2.36 2.16 4.07 3.08
8.5 8.0 7.5 7.0 6.5 6.0 5.5 5.0 4.5 4.0 3.5 3.0 2.5 2.0 1.5 1.0 0.5 0.0
f1 (ppm)

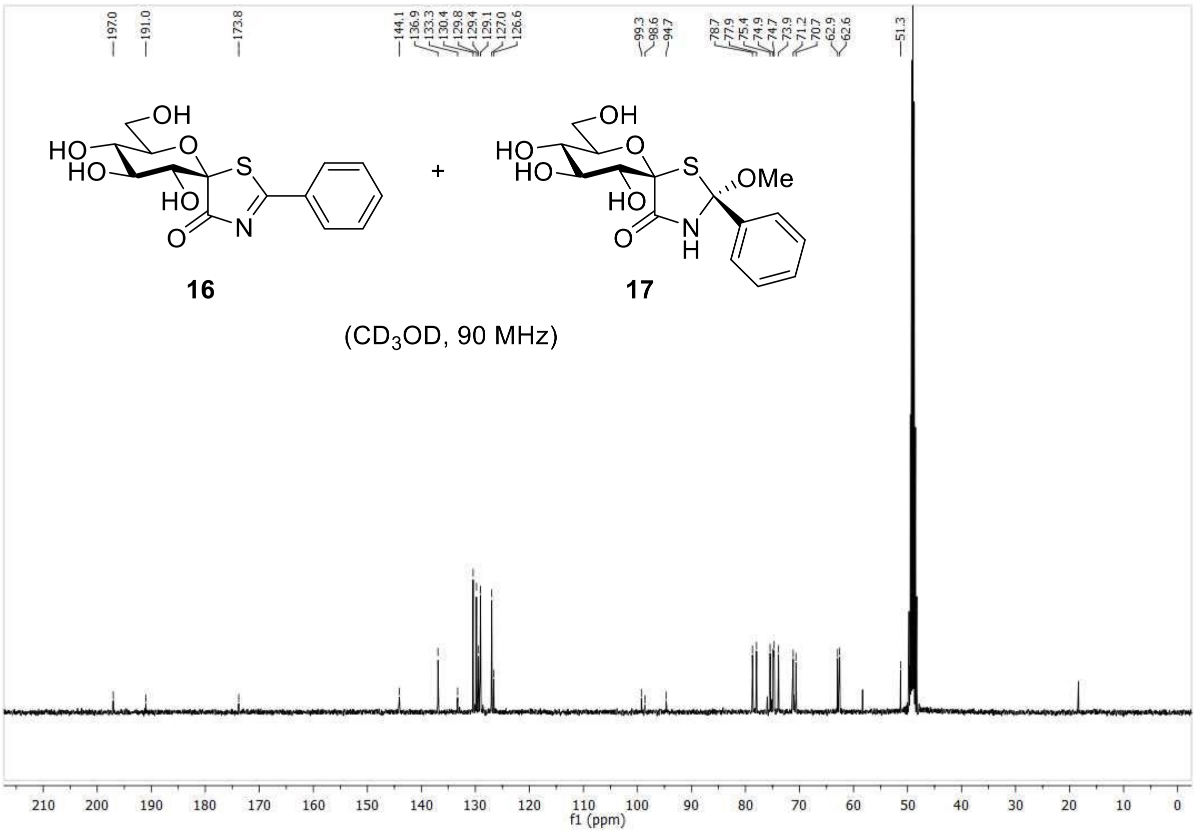

197.0 191.0 173.8
144.1 136.9 133.3 130.4 129.8 129.4 129.1 127.0 126.6
99.3 98.6 94.7
78.7 77.9 75.4 74.9 74.7 73.9 71.2 70.7 62.9 62.6
51.3
16
+
17
(CD3OD, 90 MHz)
210 200 190 180 170 160 150 140 130 120 110 100 90 80 70 60 50 40 30 20 10 0
f1 (ppm)

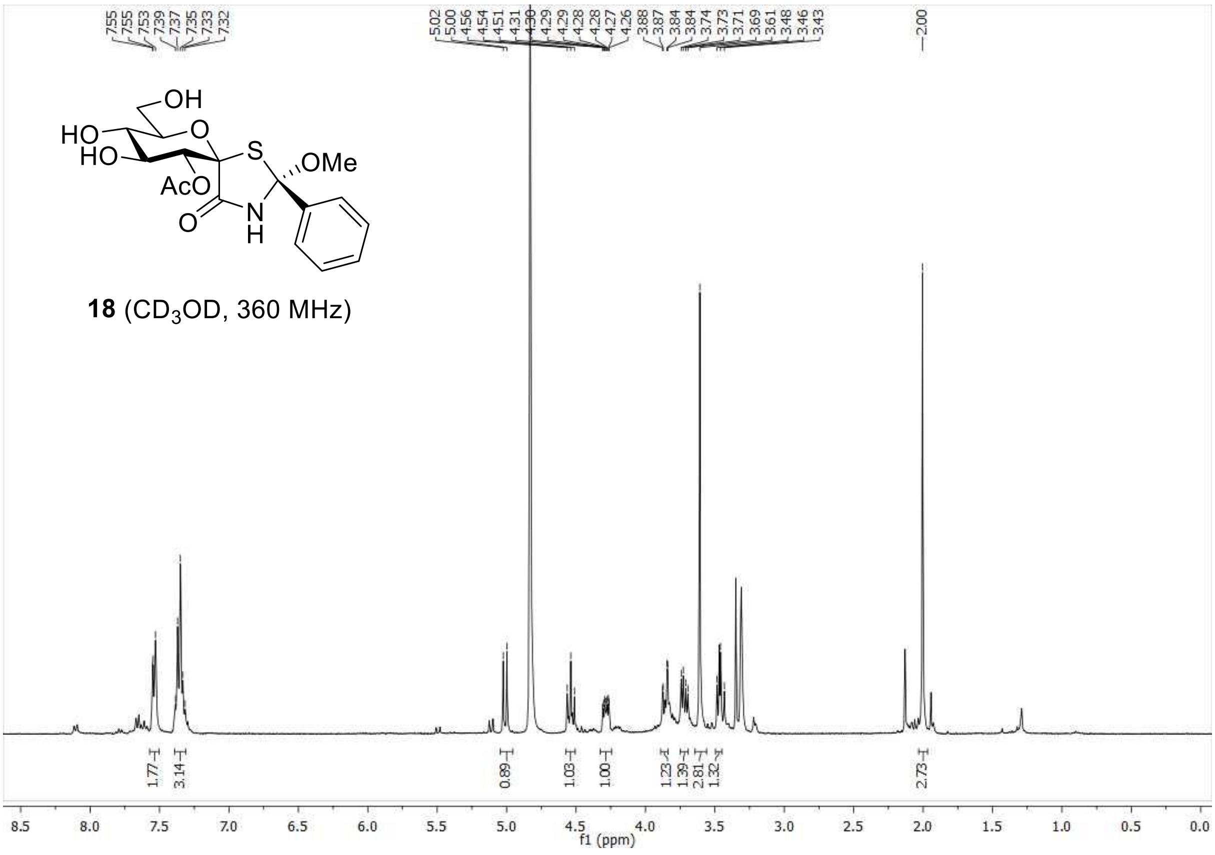

18 (CD_3OD, 360 MHz)

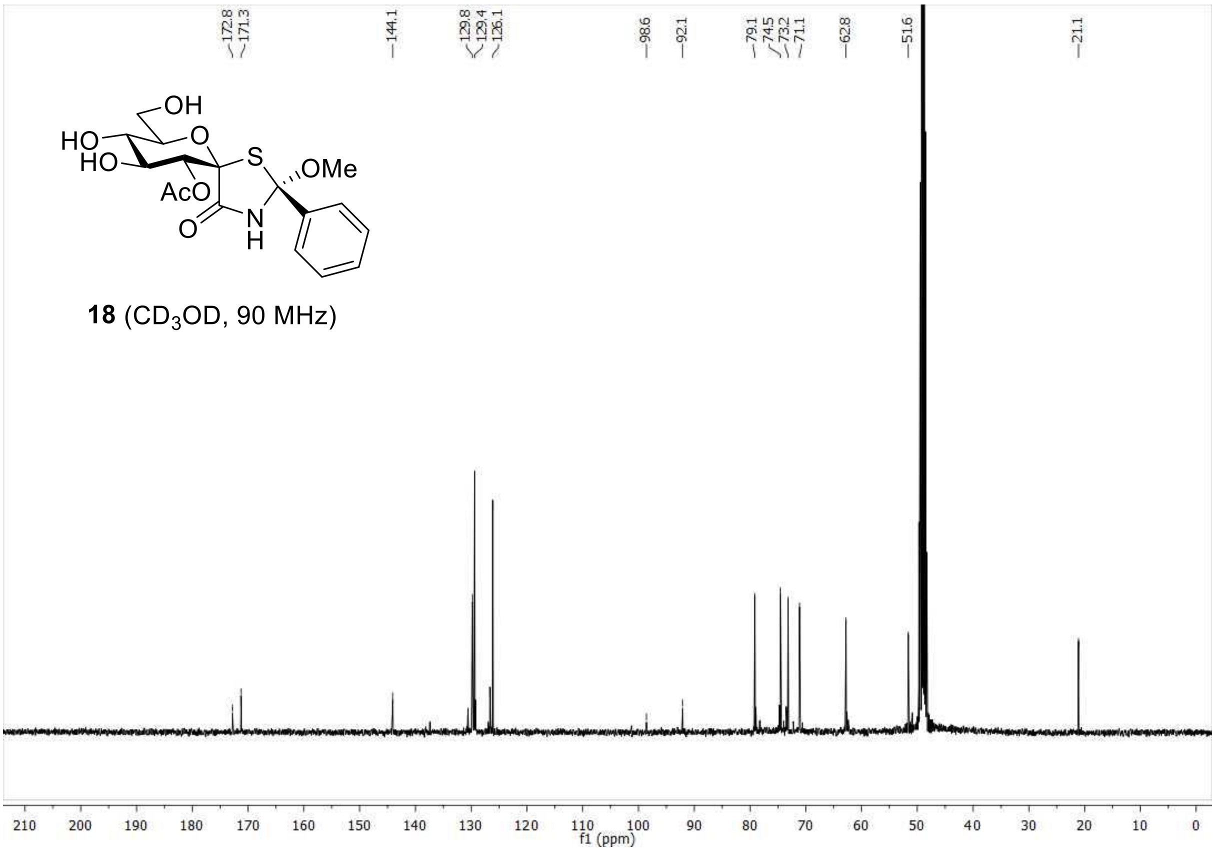

18 (CD_3OD, 90 MHz)

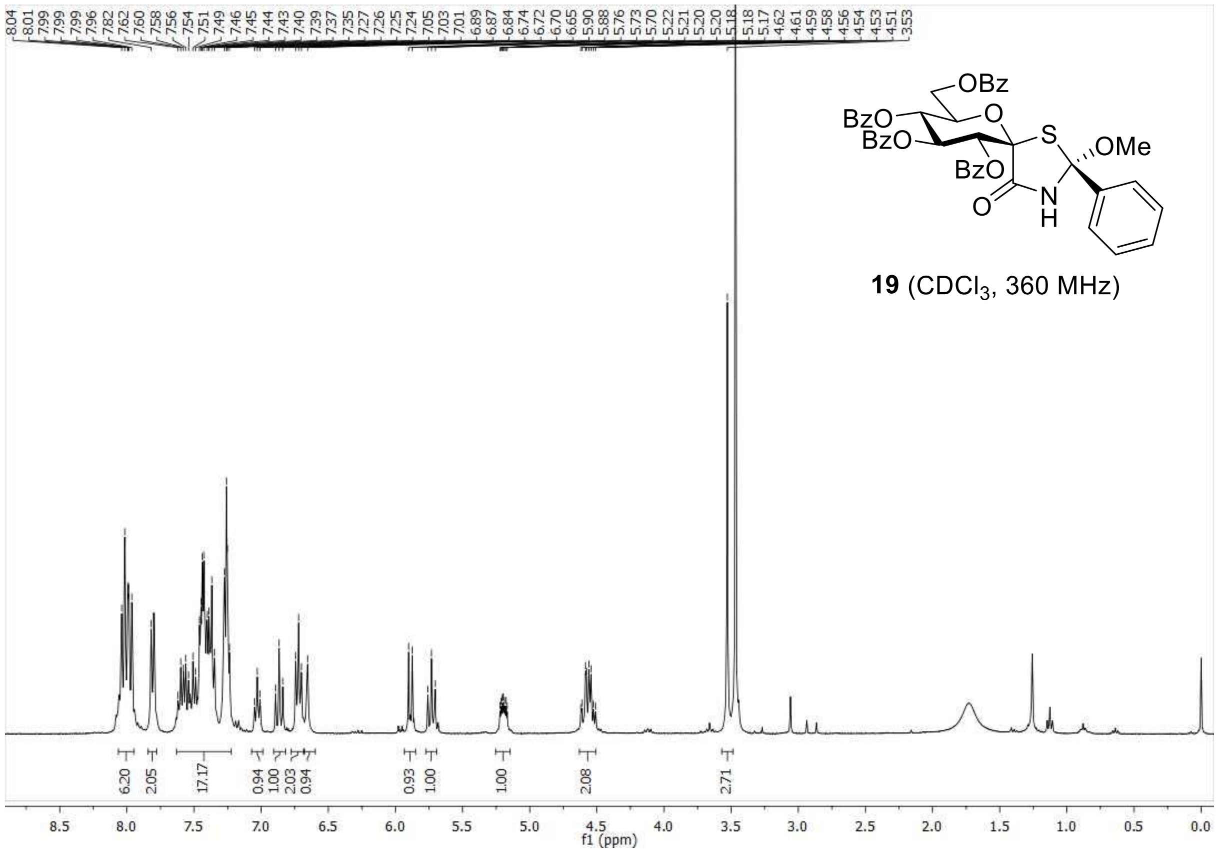
8.04 8.01 7.99 7.99 7.96 7.82 7.62 7.60 7.58 7.56 7.54 7.51 7.49 7.46 7.45 7.44 7.43 7.40 7.39 7.37 7.35 7.27 7.26 7.25 7.24 7.05 7.03 7.01 6.89 6.87 6.84 6.74 6.72 6.70 6.65 5.90 5.88 5.76 5.73 5.70 5.22 5.21 5.20 5.20 5.18 5.18 5.17 4.62 4.61 4.59 4.58 4.56 4.54 4.53 4.51 3.53
OBz
BzO
BzO
O
S
OMe
BzO
N
H
O
19 ($CDCl_3$, 360 MHz)
6.20 2.05 17.17 0.94 1.00 2.03 0.94 0.93 1.00 1.00 2.08 2.71
f1 (ppm)

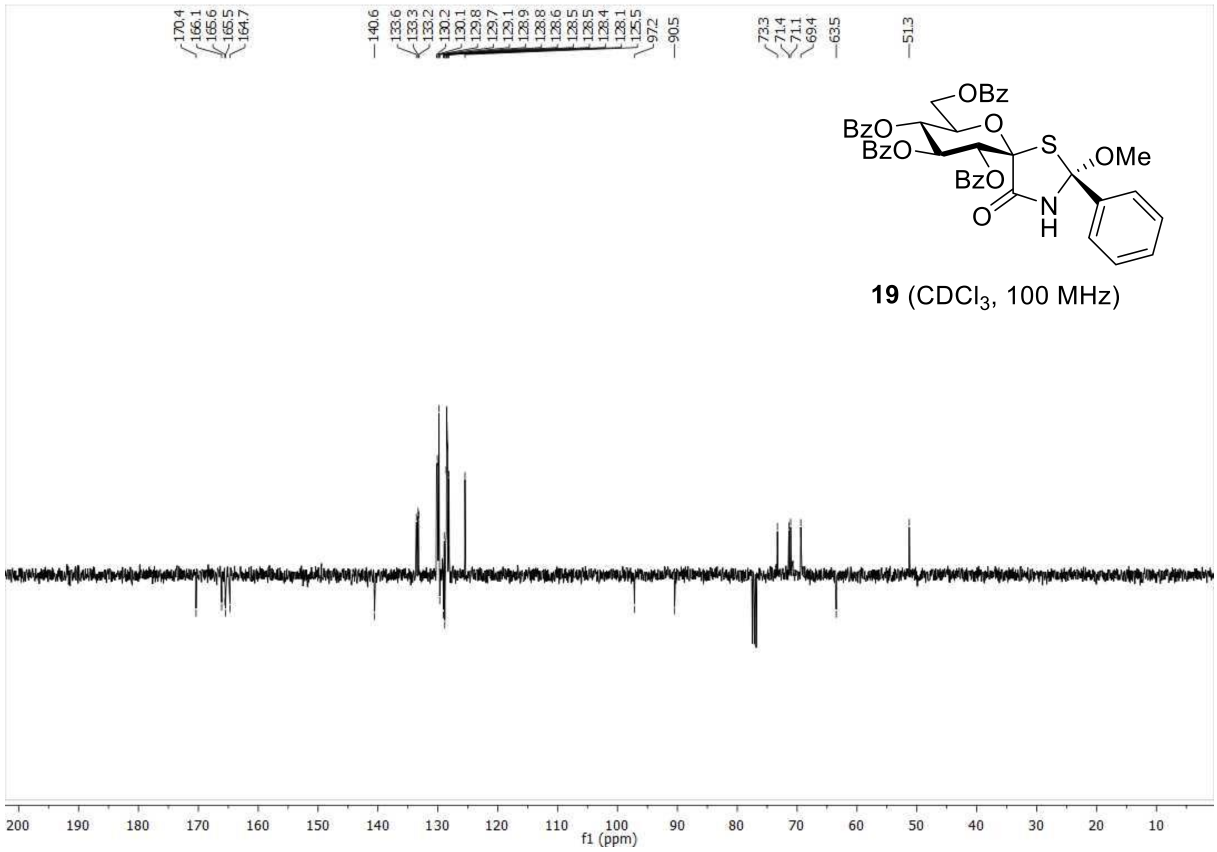
170.4 166.1 165.6 165.5 164.7 140.6 133.6 133.3 133.2 130.2 130.1 129.8 129.7 129.1 128.9 128.8 128.6 128.5 128.5 128.4 128.1 125.5 97.2 90.5 73.3 71.4 71.1 69.4 63.5 51.3
OBz
BzO
BzO
O
S
OMe
BzO
N
H
O
19 ($CDCl_3$, 100 MHz)
f1 (ppm)

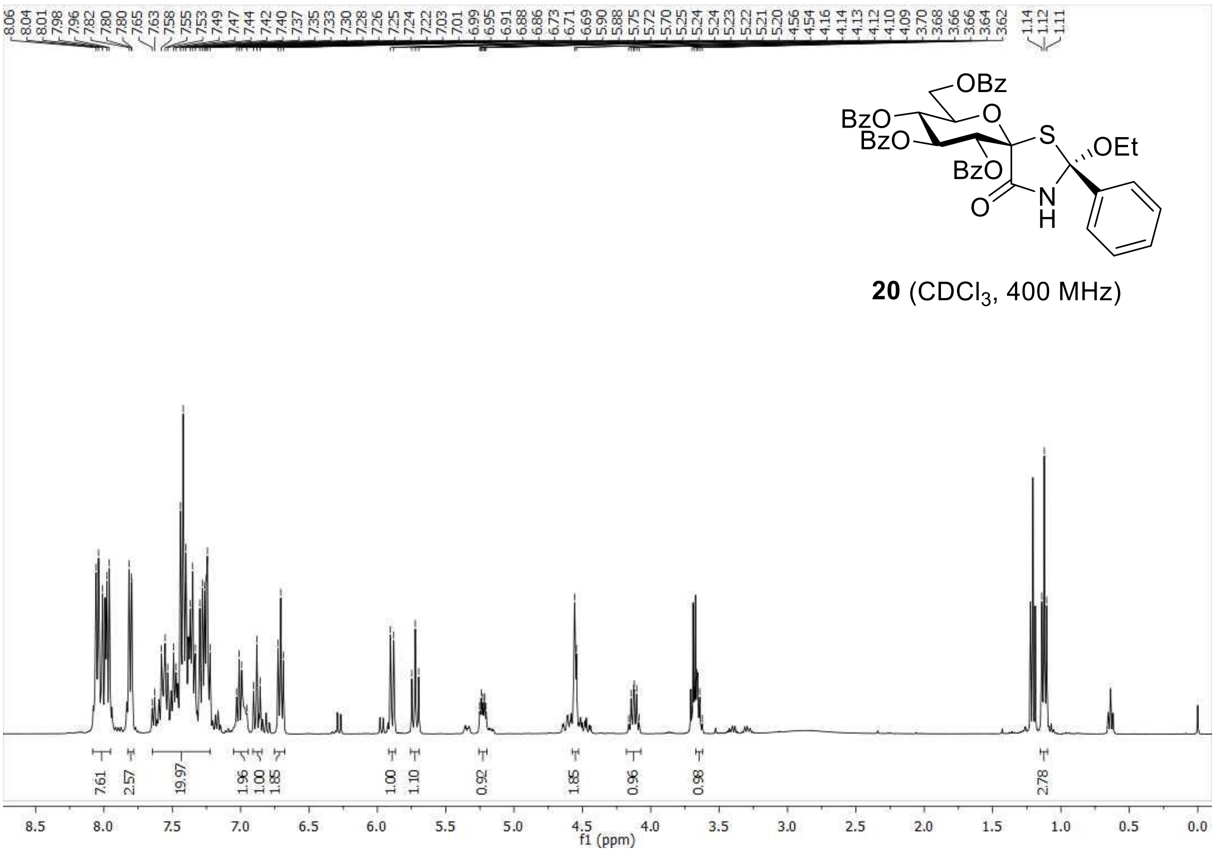
OBz
BzO
O
BzO
S
OEt
BzO
O
N
H
20 (CDCl3, 400 MHz)
f1 (ppm)

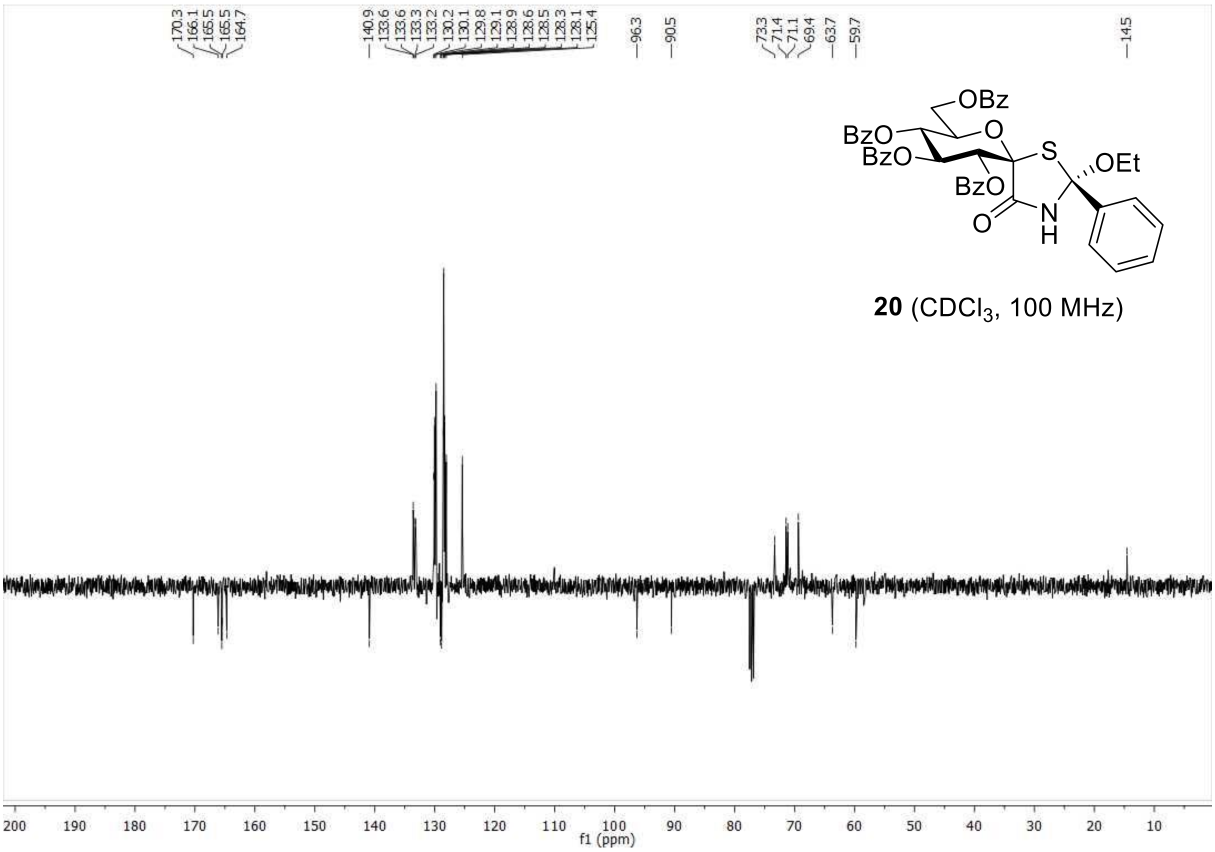
OBz
BzO
O
BzO
S
OEt
BzO
O
N
H
20 (CDCl3, 100 MHz)
f1 (ppm)

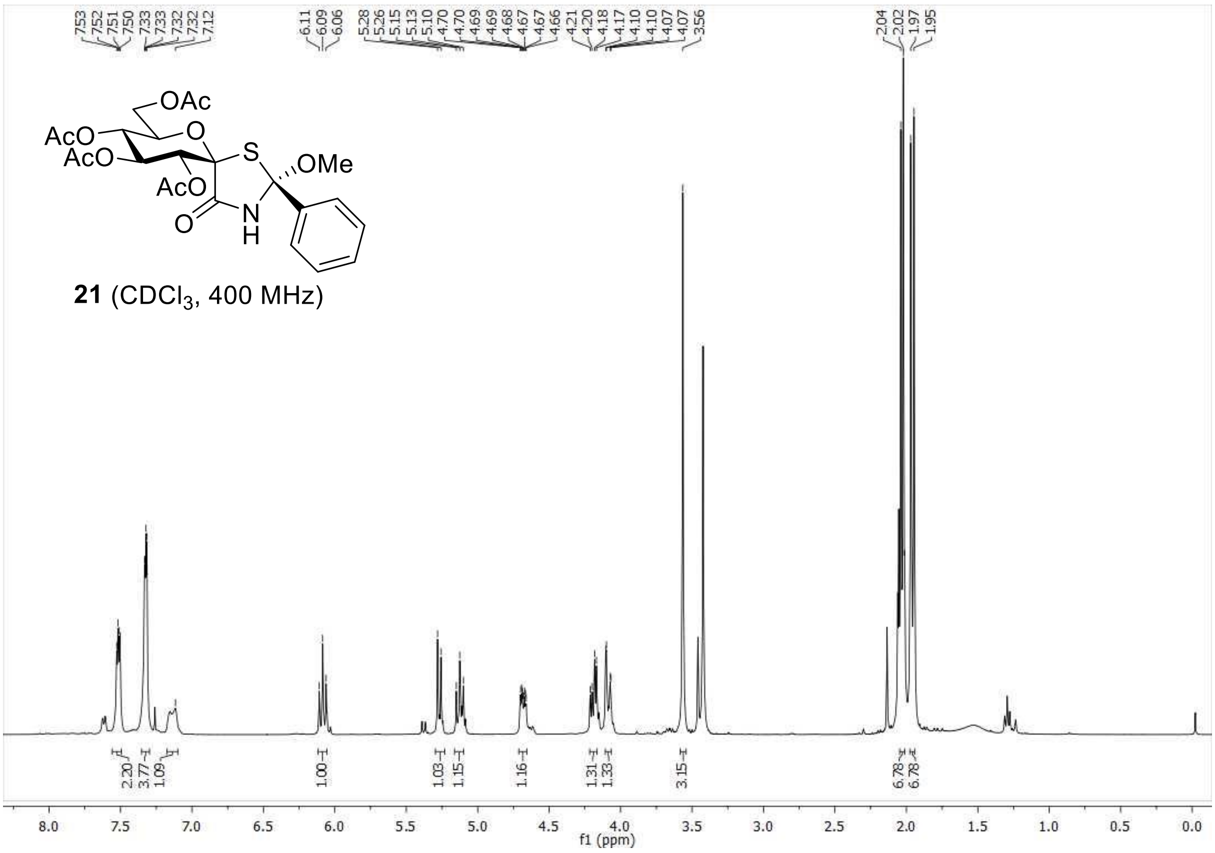

21 ($CDCl_3$, 400 MHz)

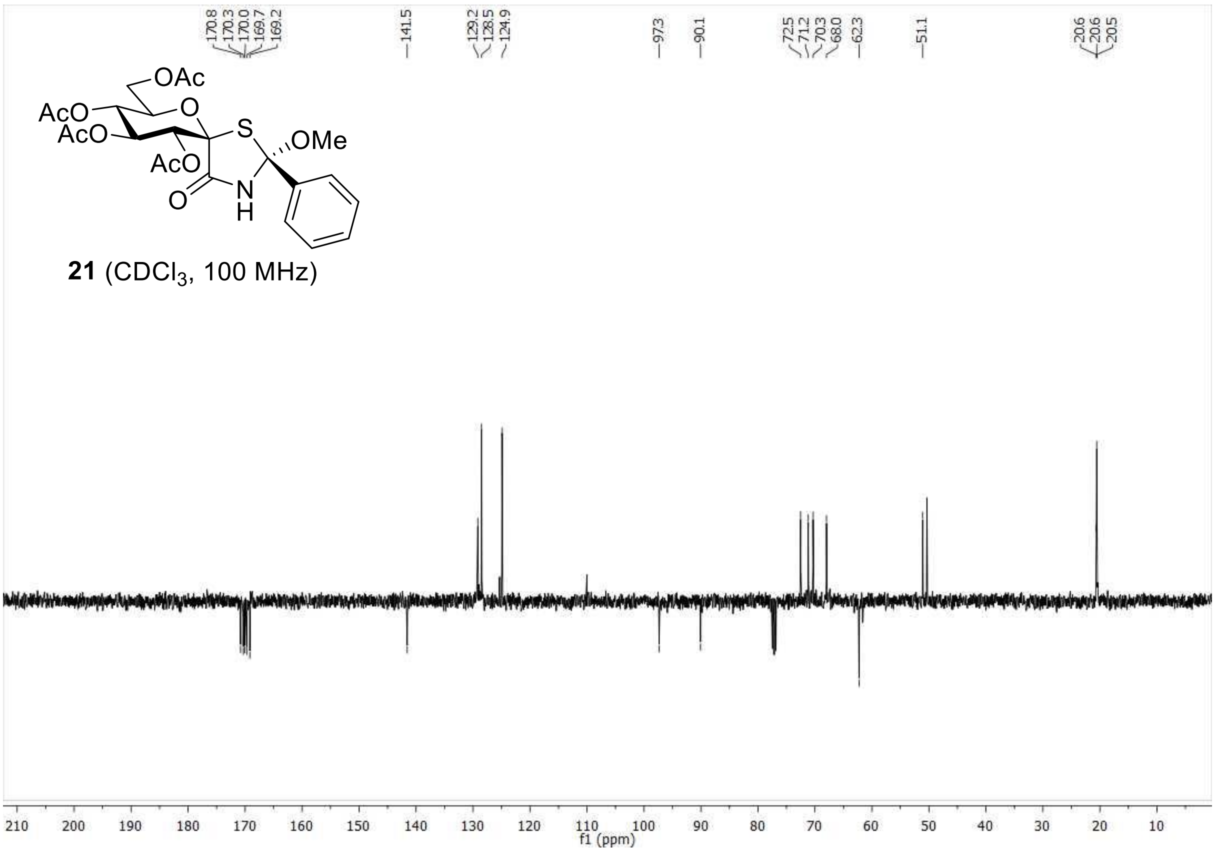

21 ($CDCl_3$, 100 MHz)

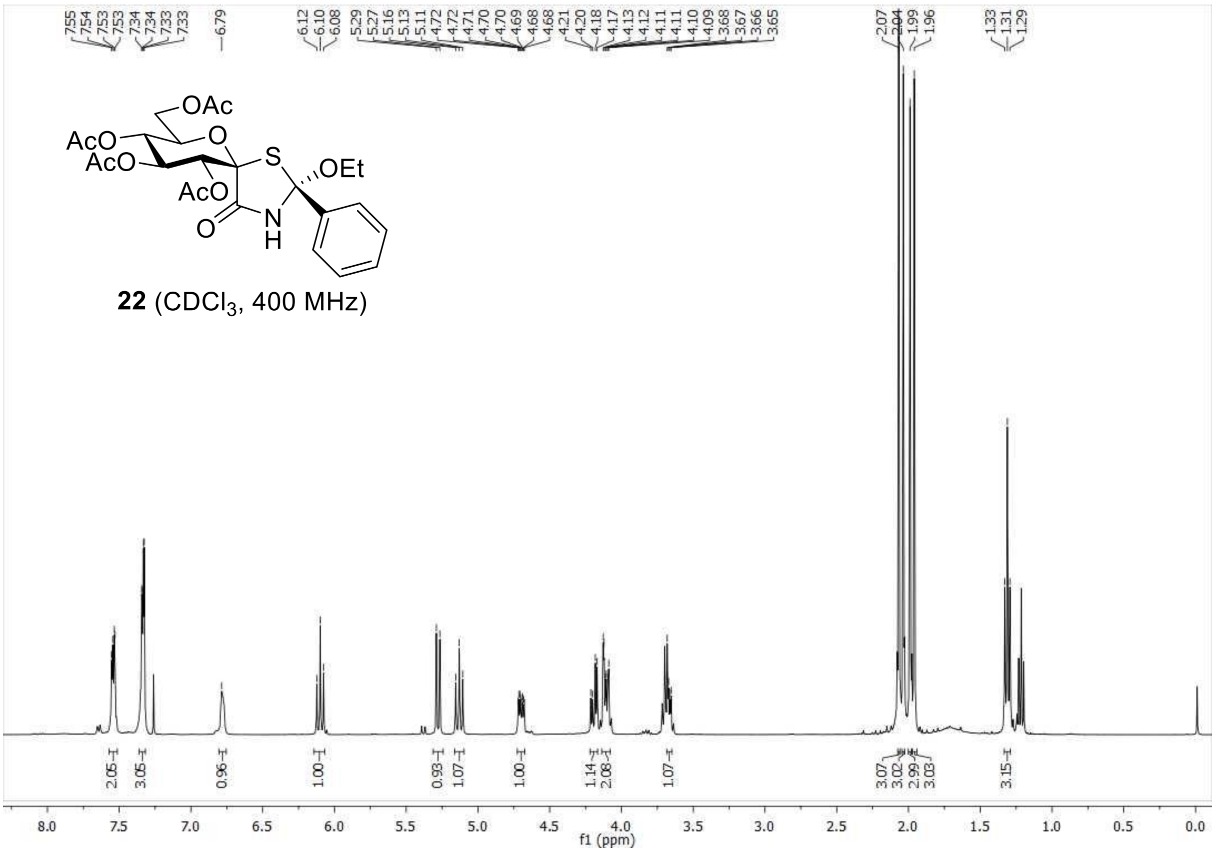

22 (CDCl$_3$, 400 MHz)
f1 (ppm)

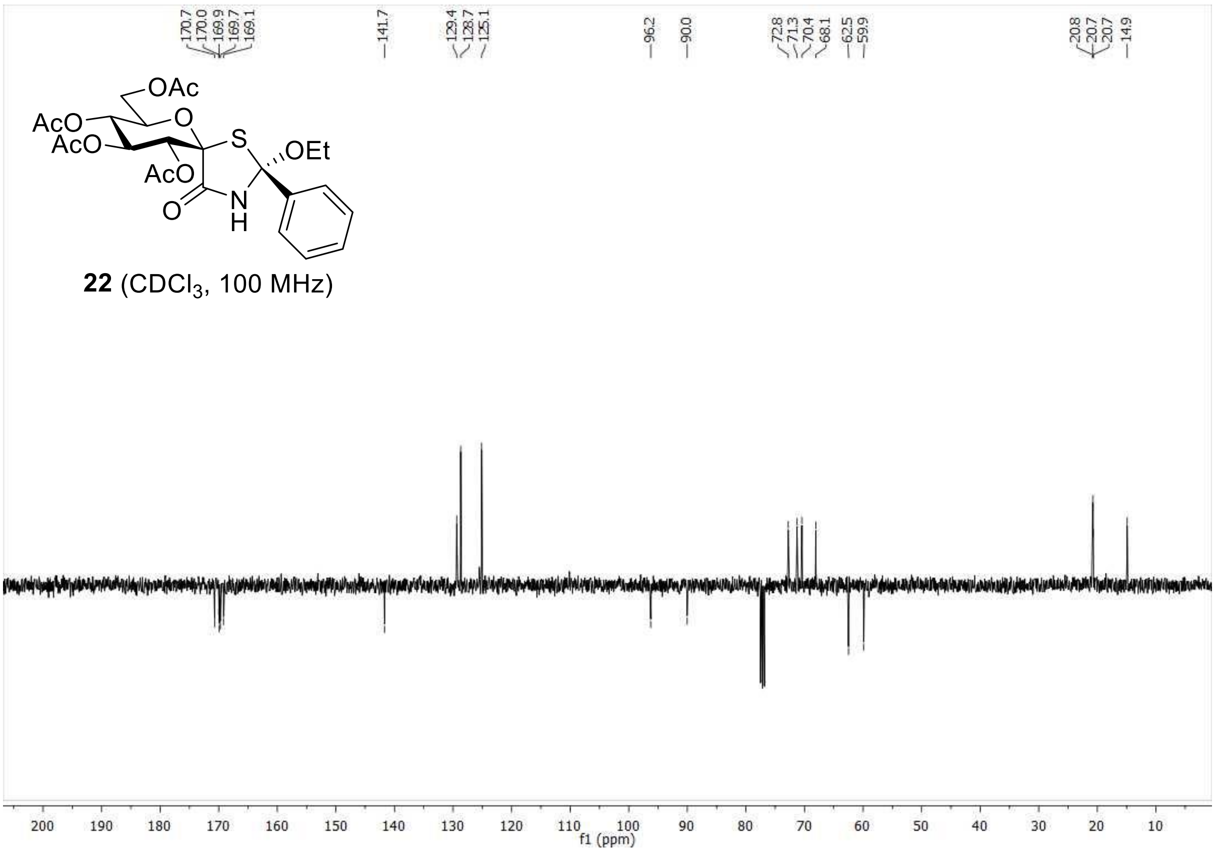

22 (CDCl$_3$, 100 MHz)
f1 (ppm)

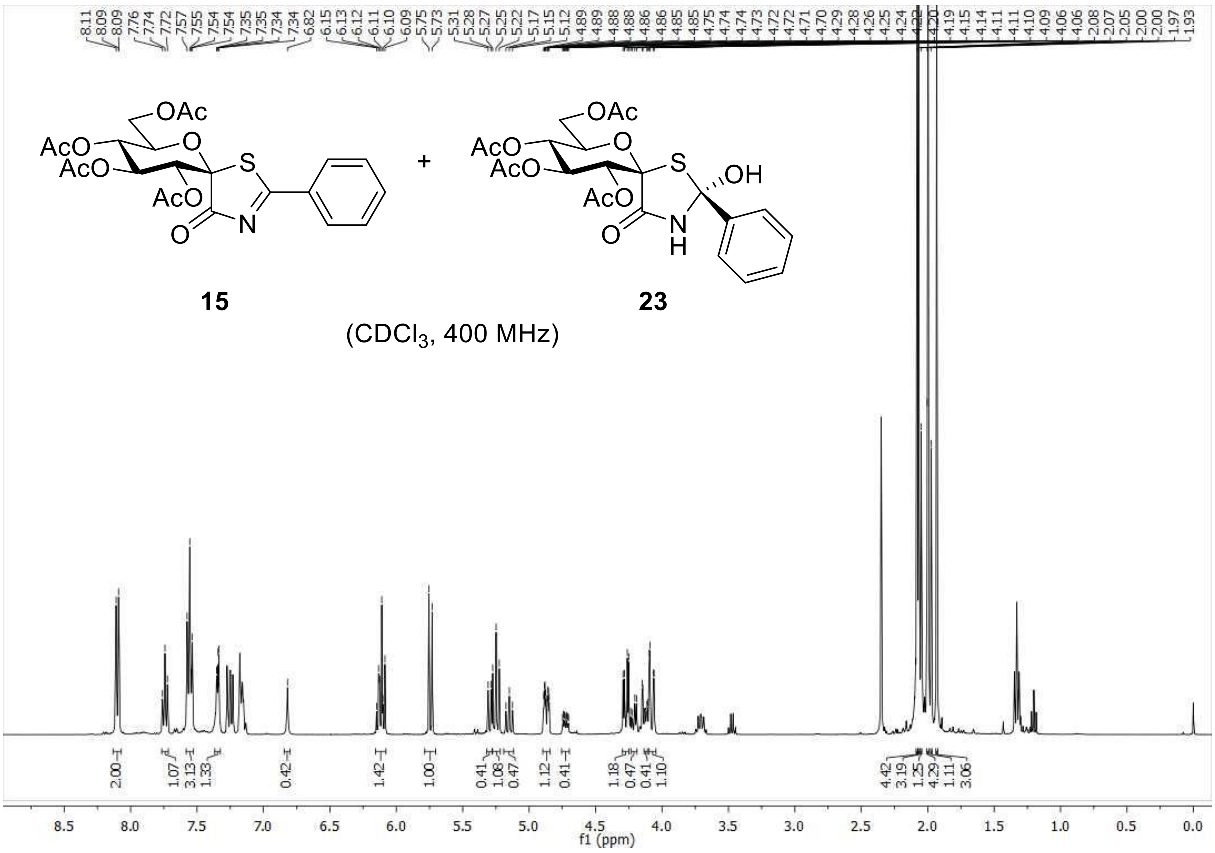

OAc
AcO
AcO
AcO
O
S
N
O
OH
H
15
+
23
(CDCl3, 400 MHz)
f1 (ppm)

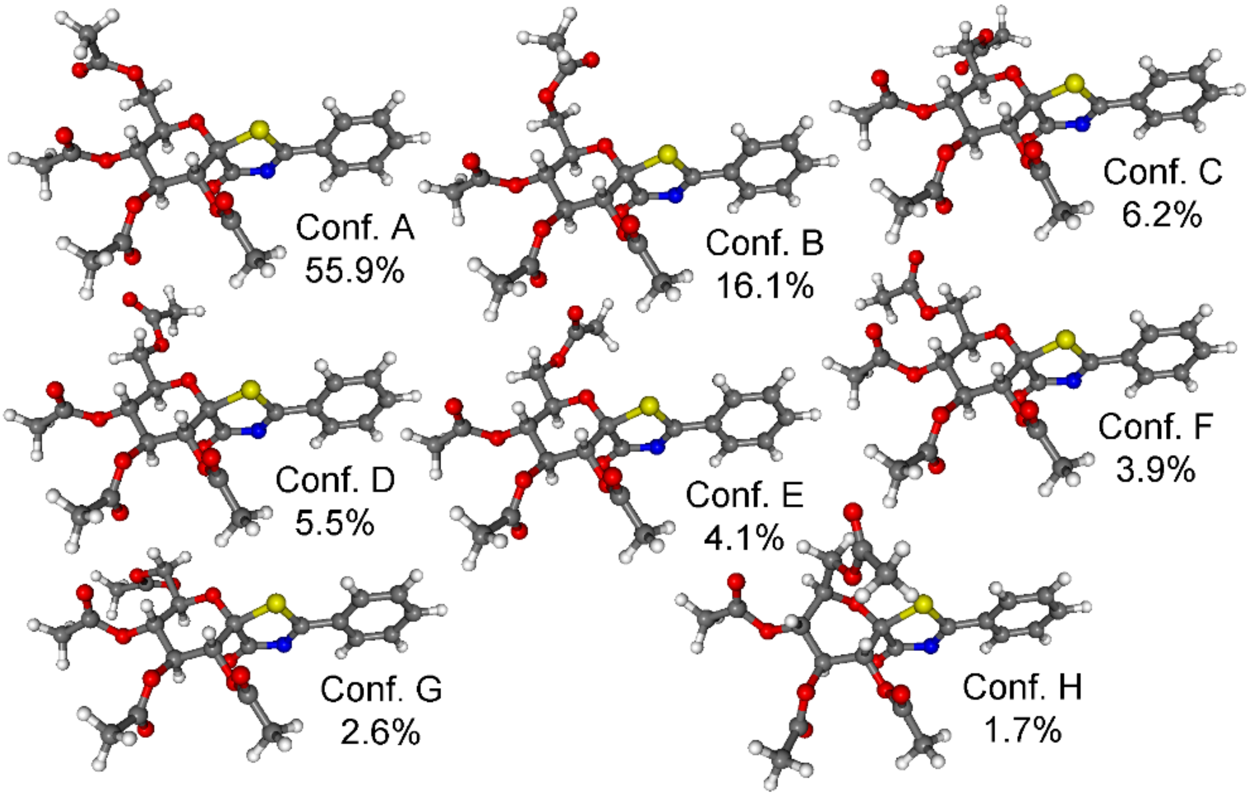

(a)

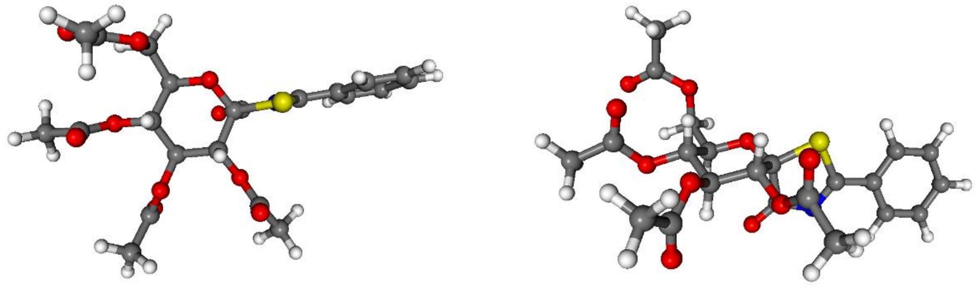

(b)

Figure S1. (a) Structures and populations of the low-energy (≥ 1%) ωB97XD/TZVP PCM/MeCN conformers of (1'*R*)-**15**; (b) different views of conformer A to show steric hindrance of the C-2 carbon by the 2'-OAc substituent.

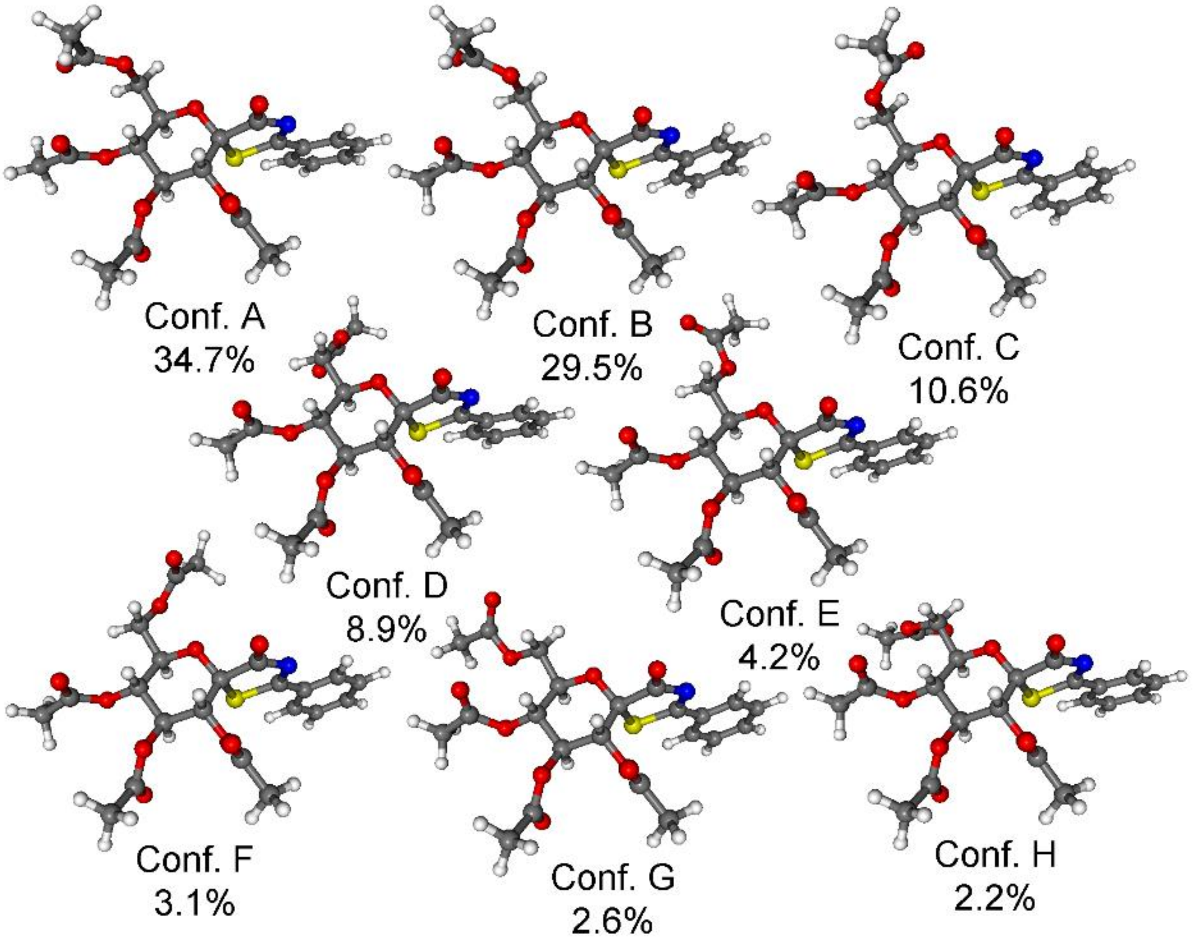

Figure S2. Structures and populations of the low-energy (≥ 1%) ωB97XD/TZVP PCM/MeCN conformers of (1'*S*)-**15**.

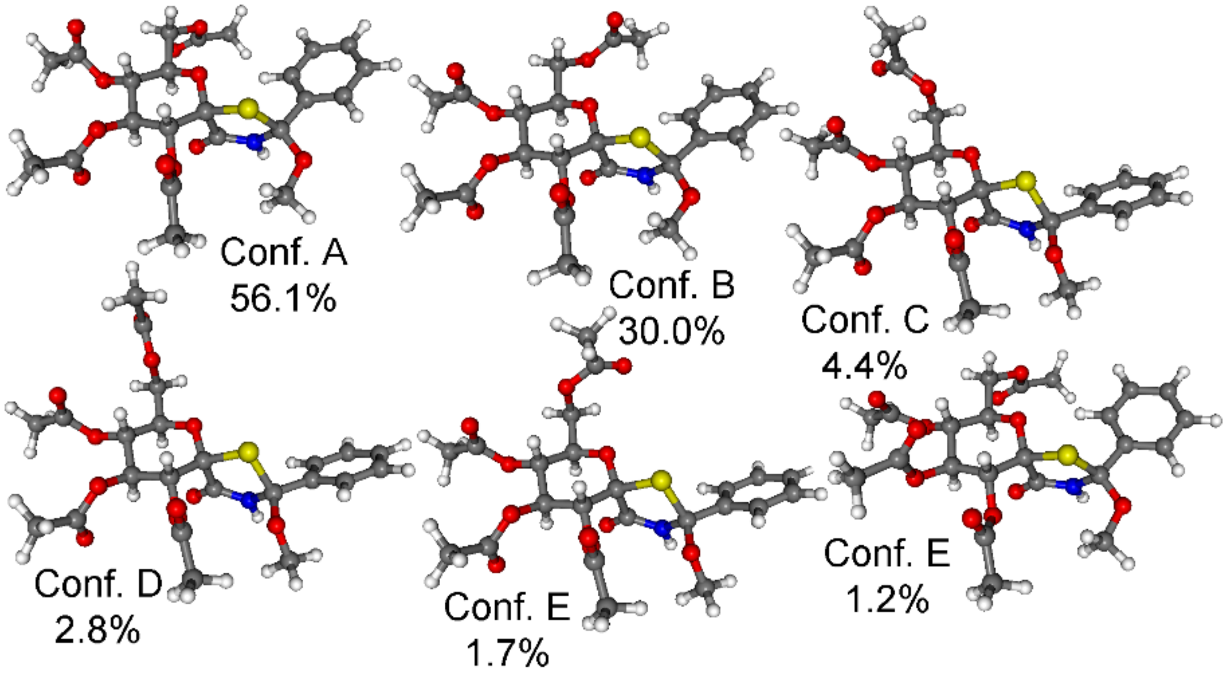

Figure S3. Structures and populations of the low-energy (≥ 1%) ωB97XD/TZVP PCM/MeCN conformers of (2*R*,1'*R*)-**21**.

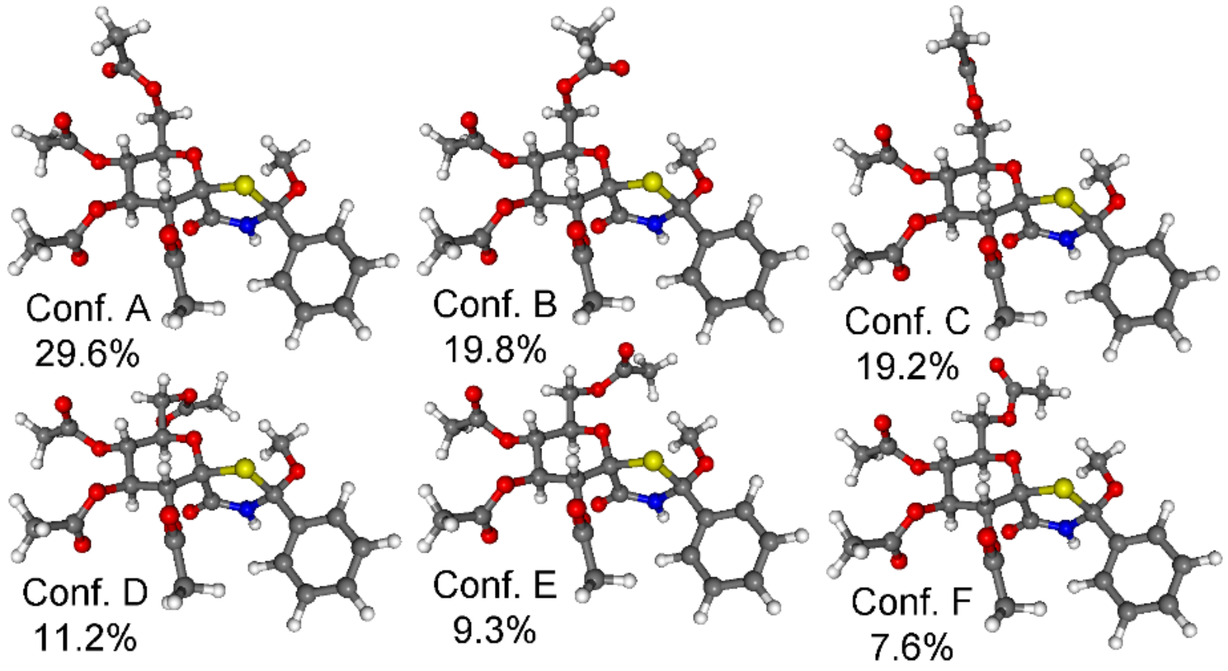

Figure S4. Structures and populations of the low-energy (≥ 1%) ωB97XD/TZVP PCM/MeCN conformers of (2*S*,1'*R*)-**21**.
